# An autopsy-based cardiac lesion evaluation system facilitates quantitative diagnosis of sudden cardiac death: development and multicenter validation of a machine learning model

**DOI:** 10.1186/s12916-025-04529-6

**Published:** 2025-11-26

**Authors:** Chao Li, Danmi Mao, Xiaohui Tan, Zhipeng Cao, Jiacheng Yue, Bing Xia, Wei Li, Donghong Liu, Weiquan Ye, Zhenyuan Wang, Yang Li, Yunle Meng, Ying Fang, Hui Yao, Shuquan Zhao, Da Zheng, Tingting Mai, Ming Zhou, Jiayi Shen, Bin Luo, Shuangbo Tang, Xiaoshan Liu, Shuiping Liu, Li Quan, Chao Liu, Erwen Huang

**Affiliations:** 1 Faculty of Forensic Medicine, Guangdong Province Translational Forensic Medicine Engineering Technology Research Center, Zhongshan School of Medicine, Sun Yat-sen University, Guangzhou, China; 2 Department of Clinical Medicine, Zhongshan School of Medicine, Sun Yat-sen University, Guangzhou, China; 3 Guangzhou Key Laboratory of Forensic Multi-Omics for Precision Identification, School of Forensic Medicine, Southern Medical University, Guangzhou, China; 4 Department of Forensic Pathology, School of Forensic Medicine, China Medical University, Shenyang, China; 5 College of Forensic Medicine, Guizhou Medical University, Guiyang, China; 6 Department of Medical Ultrasonics, The First Affiliated Hospital of Sun Yat-sen University, Guangzhou, China; 7 Jinan University Center of Forensic Science, Guangzhou, China; 8 Department of Forensic Pathology, College of Forensic Medicine, Xi’an Jiaotong University, Xi’an, China; 9 Department of Forensic Pathology and Anthropology, Institute of Forensic Science, The Ministry of Public Security, Beijing, China; 10 Key Laboratory of Forensic Pathology, Ministry of Public Security, Guangzhou, China; 11 National Anti-Drug Laboratory Guangdong Regional Center, Guangzhou, China

**Keywords:** Sudden cardiac death, Forensic autopsy, Cardiac pathology, Machine learning, Myocardial infarction

## Abstract

**Background:**

The clinical prevention and forensic diagnosis of sudden cardiac death (SCD) remain challenging due to the absence of standardized quantitative criteria for evaluating cardiac morphological and histopathological alterations. We aimed to develop a machine learning-driven cardiac lesion evaluation system to facilitate quantitative diagnosis and risk stratification of SCD.

**Methods:**

A total of 2284 adult autopsy cases from the forensic center at Sun Yat-sen University and 1883 external cases from five independent centers were enrolled to develop and validate an autopsy-based diagnostic model. Eight machine learning algorithms were employed, with the optimal model further tested in human–machine collaborative experiments. The model was subsequently transformed to identify myocardial infarction in a prospective clinical cohort of 204 patients presenting with chest pain.

**Results:**

SCD cases exhibited significantly greater right ventricular wall thickness (OR: 1.17 [95% CI: 1.04–1.32] per mm) and larger valve annulus circumferences, including tricuspid (OR: 1.17 [95% CI: 1.04–1.33] per cm), pulmonary (OR: 1.54 [95% CI: 1.34–1.76]), mitral (OR: 1.16 [95% CI: 1.04–1.29]), and aortic (OR: 1.24 [95% CI: 1.06–1.44]) valves. The logistic regression model demonstrated strong discriminatory performance for SCD, achieving an area under the receiver-operating characteristic curve (AUC) of 0.839 (95% CI: 0.821–0.858) in the training set and 0.840–0.907 across external validation cohorts. Pathologists assisted by the model showed improved diagnostic accuracy, with higher AUC (*P* = 0.004) and sensitivity (*P* = 0.01) for SCD diagnosis. For sudden coronary artery death, the morphology-based model achieved an AUC of 0.781 (95% CI: 0.738–0.825), while its performance in detecting myocardial infarction using echocardiography-measurable features yielded an AUC of 0.697 (95% CI: 0.587–0.817).

**Conclusions:**

This rigorously validated model serves as a novel assistant tool for pathologists to achieve quantitative diagnosis of SCD and provides clinicians with a potential tool for myocardial infarction identification and SCD warning.

**Graphical Abstract:**

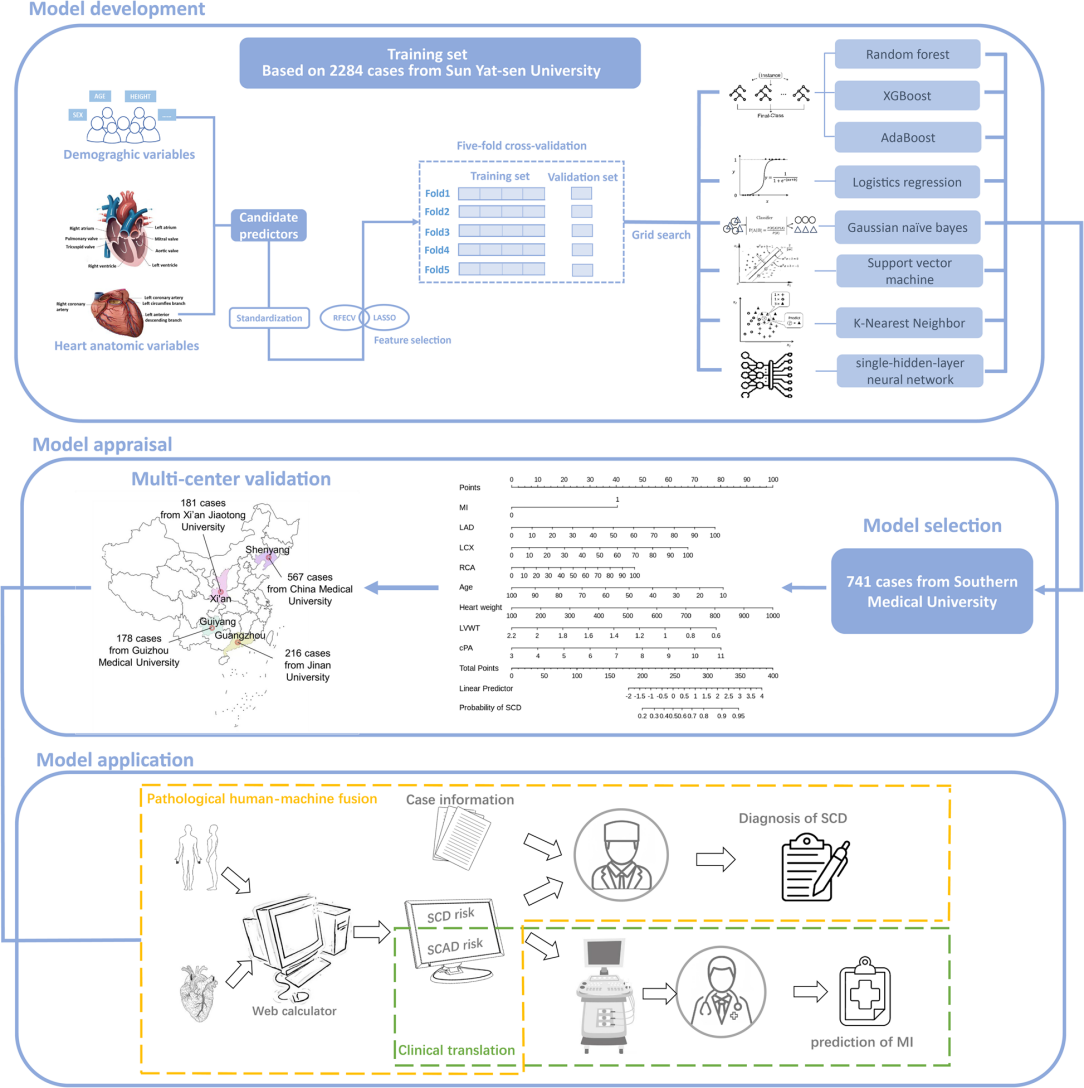

**Supplementary Information:**

The online version contains supplementary material available at 10.1186/s12916-025-04529-6.

## Background

Sudden cardiac death (SCD) represents the terminal manifestation of diverse cardiovascular disorders and accounts for millions of deaths worldwide annually and poses a significant public health challenge [1]. For the cardiac etiologies, severe coronary artery disease (CAD) is reported to be the fatal cause of approximately 80% of SCD, following by non-ischemic cardiomyopathies, cardiac inflammation, congenital heart disorders, valvular heart diseases and electrical heart diseases [2, 3].

Approximately 50% of SCD victims experience sudden death as the first clinical manifestation of previously undiagnosed heart disease, which presents significant challenges for both emergency intervention and postmortem diagnosis, particularly in cases lacking premortem records or reliable witness accounts [4, 5]. Furthermore, the forensic determination of SCD differs from clinical diagnosis, which is primarily based on the electrocardiogram, imaging and biomarkers reflecting early myocardial injury [6]. The diagnostic utility of established biochemical markers is limited in postmortem settings due to tissue autolysis, microbial degradation, and metabolic changes, particularly with delayed sample collection [7–9]. Although various biochemical markers have been uncovered to indicate the occurrence of SCD, including mRNAs, circular RNAs, immunohistochemical biomarkers, proteins, and metabolites [10–14], these emerging biomarkers are still far from application, not only due to the general lack of external validation for these pilot researches, but also for the high requirements of detection methods and high costs.

Therefore, the forensic determination of SCD still depends on the systematic autopsy, including the visual measurements of cardiac macro-morphological features and histopathological staining examinations, combined with available medical records and witness information and differential examinations [15]. Nevertheless, the lack of unified evaluation criteria on the risk factors and lesions poses the risk of inconsistent diagnoses which was made according to the subjective experience of different experts.

In this multicenter study, we aimed to construct and externally validate an autopsy-based machine learning (ML) framework for systematic cardiac lesion evaluation, to provide an objective determination of SCD and assist the accurate diagnosis in the widest forensic practice. As CAD accounts for the majority of SCD [2, 3] and represents cardiac morphological alternations [16–19], we also tried to transform the autopsy-based model to be clinically available and evaluate the clinical relevance of the model for myocardial infarction (MI) identification.

## Methods

### Study design

Non-criminal death cases were consecutively enrolled across the forensic medicine centers in six universities: Sun Yat-sen University (SYSU), Southern Medical University (SMU), Jinan University (JNU), China Medical University (CMU), Guizhou Medical University (GMU), and Xi’an Jiaotong University (XJTU). The inclusion criteria were as follows: (1) age ≥ 16 years old; (2) completion of systematic forensic autopsy including macroscopic, histopathological, biochemical, and toxicological examinations with preserved specimens. The death causes of each corpse were determined by no less than three professional forensic pathologists (at least one senior expert) and checked by another senior pathologist, according to the findings in the systematic autopsy, information of death circumstances and available medical history. SCD was defined as unforeseen sudden natural death from cardiac etiologies within a short period from the onset of symptoms, with postmortem cardiac lesions observed and with non-cardiac fatal causes obviated. Specifically, sudden coronary artery death was defined as SCD caused by CAD. A few cases were excluded when the death causes remained unclear due to insufficient information about exclusive diagnosis or lack of complete autopsy procedure, or the cardiac morphological records were unavailable.

We also conducted a prospective clinical cohort that consecutively recruited patients who suffered from chest pain and received coronary angiography or CT angiography, with echocardiography performed around in the First Affiliated Hospital of Sun Yat-sen University, from April to September, 2024, to explore the correlation between heart morphological features and MI. MI was detected based on the clinical symptoms, electrocardiogram test, serum myocardial injury biomarkers and coronary angiography.

The study conformed to the principles outlined in the Declaration of Helsinki and was approved by the Medical Ethics Committee of Sun Yat-sen University (Approval No. 2024–062) and the First Affiliated Hospital of Sun Yat-sen University (Approval No. 2024–599). Informed consents were obtained from the bereaved of victims in forensic centers and patients in the clinical cohort. The reporting of this study follows the STrengthening the Reporting of OBservational studies in Epidemiology (STROBE) and Transparent Reporting of a multivariable prediction model for Individual Prognosis Or Diagnosis (TRIPOD) + artificial intelligence guideline [20, 21].

### Candidate predictors

The heart examination was performed in a standard inflow-outflow procedure in accordance with the Forensic medicine–General technical specifications for examination of death (GA/T 147–2019), and the Forensic medicine–Specifications for examination of sudden death (GA/T 170–2019), the public safety industry standard of the People’s Republic of China. A total of fifteen demographic and macro-morphological features were derived from forensic pathology reports and included in the candidate variable set. The demographic information comprised age, sex, body height, and abdominal subcutaneous fat thickness. We included seven morphological features of heart that are routinely measured in forensic practice, including heart weight, left ventricle wall thickness (LVWT), right ventricle wall thickness (RVWT) and circumference of the tricuspid annulus (cTA), pulmonary annulus (cPA), mitral annulus (cMA), and aortic annulus (cAA). The stenosis degrees of left anterior descending artery (LAD), left circumflex artery (LCX) and right coronary artery (RCA) were measured as continuous variables while MI was detected based on the gross and histopathological characteristics [22] and considered as a dichotomous variable. Details about the heart examination were shown in Additional file 1: Supplementary methods.

### Work flow of the model development

**Fig. 1:**
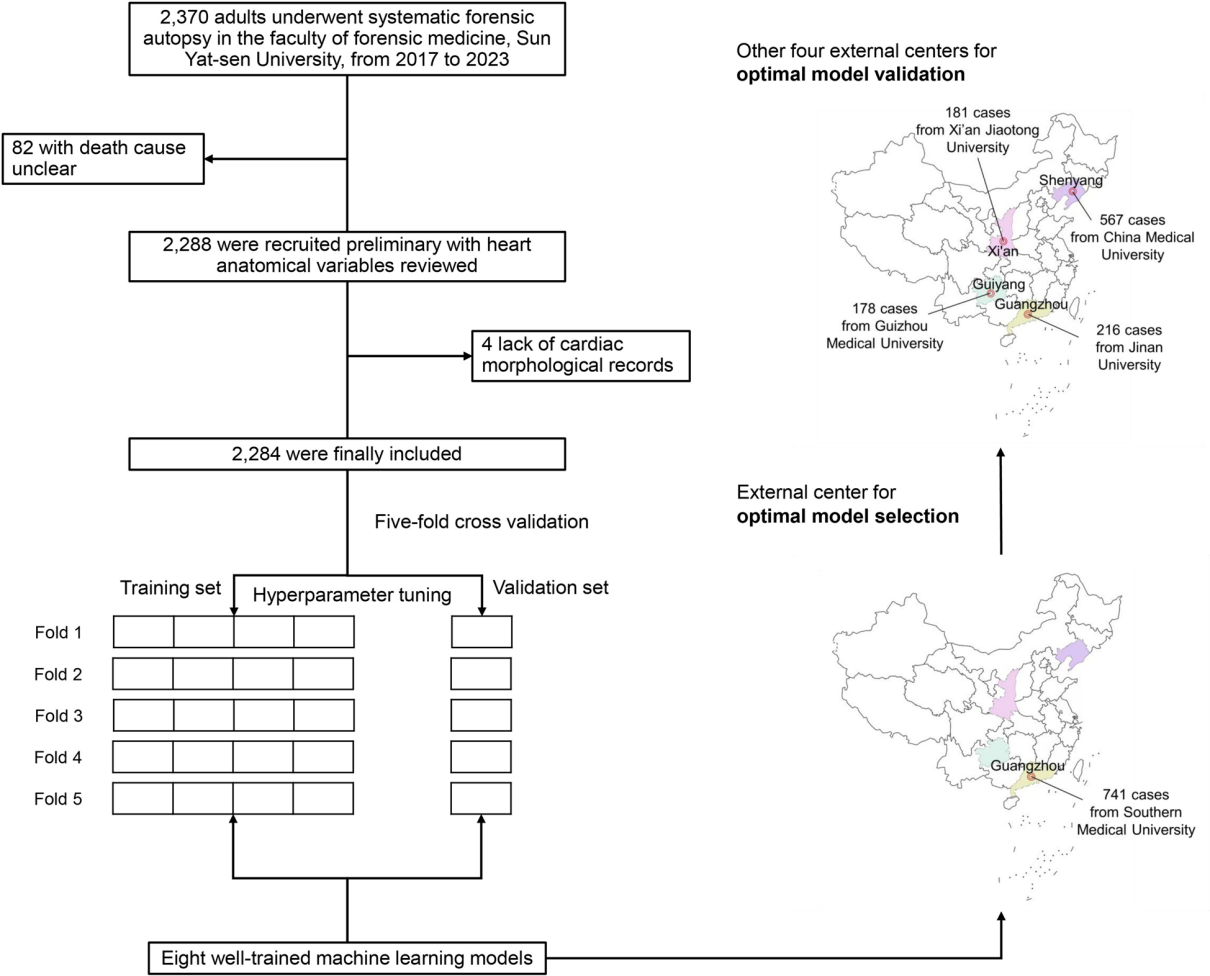
Study flowchart and machine learning model development. A total of 2284 cases in the Sun Yat-sen University dataset and 1883 in theSouthern Medical University, China Medical University, Jinan University, Xi'an Jiaotong University, and Guizhou Medical University datasets (with 30.0% and 23.4% diagnosed SCD, respectively) were recruited to develop and externally validate the machine learning models

As shown in Graphic abstract and Fig. 1, eight ML algorithms were applied to construct the diagnostic models for identifying SCD in the SYSU dataset (training set). The well-tuned ML models were initially evaluated in the SMU center to elect the optimal model, which was then externally validated in CMU, JNU, GMU, and XJTU centers.

#### Data processing

Missing values in datasets were independently imputed using a non-parametric missForest technique, which is demonstrated to be a robust method that can address the missing issue in mixed-type and high-dimensional dataset through an iterative imputation scheme [23]. Standardization was performed for continuous variables in the imputed training set and the mean value along with standard deviation was then applied to standardize other datasets.

#### Feature selection

Two distinct techniques, the least absolute shrinkage and selection operator (LASSO) method and recursive feature elimination based on random forest (RF-RFE), with tenfold cross-validation were adopted for the feature selection in the training set. The intersection of these two methods was employed for the model construction. The multicollinearity was diagnosed using variance inflation factor and the correlation heatmap. SHAP (Shapley Additive exPlanations) values based on extreme gradient boosting trees (XGBoost) algorithm were applied to improve the local and global interpretability of machine learning models and utilized for ranking feature importance [24].

#### Model construction

Eight ML algorithms, including random forest, XGBoost, adaptive boosting trees, logistic regression (LR)-nomogram, support vector machine with radial basis function kernel, *K*-nearest neighbor, Gaussian naïve bayes, and single-hidden-layer neural network—were implemented for model development in the training set. Grid search with fivefold cross-validation strategy was utilized for hyperparameter tuning. Models with the optimal combination of hyperparameters were primarily evaluated in SMU center. The model yielding the highest area under the receiver-operating characteristic curve (AUC) was elected as the final prediction model, with an interactive online calculator compiled via *ShinyApp* (the URL for the web calculator is: https://lich283.shinyapps.io/ML_model_forensic_diagnosis_SCD_sysu/). The generalizability of the prediction model was further tested in CMU, JNU, GMU and XJTU, on behalf of cases from northeastern, southern, middle and northwestern China.

### Model performance appraisal

The model performance was comprehensively appraised in the training set and externally in other five representative centers. Receiver-operating characteristic (ROC) curve, as well as the AUC, were employed to appraise the discrimination ability. Moreover, statistical metrics, including accuracy, sensitivity, specificity, positive predictive value, negative predictive value, *F*1 score and Kappa value, were calculated based on the cut-off value that achieved the maximum Youden’s index in the training set.

### Human–machine fusion

As the model acted as a severity evaluation system of cardiac lesions, with death circumstance information and competitive fatal factors not considered, we intended to propose a human–machine fusion manner to facilitate the forensic practice. To test the potential of such a work pattern, we invited four senior and four junior forensic pathologists, with more than 10 years and 3 years of experience, respectively, to participate in the human–machine fusion experiment. The external XJTU dataset was selected as the experiment set, which was concealed from all pathologists before the experiment. All cases were independently reviewed by participating pathologists who rendered binary diagnoses based on available information excluding circumstantial evidence, thereby simulating real-world diagnostic challenges in forensic practice. The discrimination capacity between each pathologist and model was compared according to AUC using Delong’s test and sensitivity and specificity using McNemar test. After the human–machine comparison, the predicted probabilities about the SCD diagnosis for each case, along with the overall performance metrics of the developed ML model in both the training set and other external centers (except for the experiment center), were disclosed to the pathologists so that they were able to re-consider their diagnoses and freely made a decision whether to change their initial diagnosis. One-tailed Wilcoxon signed-rank tests were conducted to compare the differences of AUC, sensitivity and specificity of pathologists before and after the model assistant.

### Clinical translation

An interdisciplinary translation, from forensic identification of sudden coronary artery death among individuals with CAD and natural death, to clinical identification of MI among patients with chest pain, was conducted to test the predictive potential of macro-morphological features of heart and ML models. The same feature selection approaches were applied and the selected morphological variables in forensic setting were transformed to be clinically available: the circumferences of heart valves were transformed to the diameters that could be measured using two-dimension cardiac ultrasound according to the practice guidelines [25–27]. Thickness of left ventricular posterior wall and right ventricular anterior wall at end diastole were measured in parasternal 2D-long axis view [27]. A LR model including the converted variables was applied to construct the identification model of MI.

### Statistics analysis

Categorical variables were summarized as number with frequency while continuous variables were displayed by mean with standard deviation or median with interquartile ranges, depending on their distribution. Univariate and multivariate-adjusted LR were utilized to explore the relationship between each candidate variable and SCD. All statistics analyses, model development, and performance evaluation of the ML models were conducted using R software (version 4.3.2).

## Results

### Study population characteristics

Of 2370 initially screened cases in the SYSU cohort (2017-2023), 2284 were finally included in this study (Fig. 1), with 73.7% male predominance and a mean age at death of 46.8 (standard deviation: 14.9) years. 73.2% of the included individuals were dead of natural causes and 52.8% were dead in a sudden manner. Five hundred eight-one (34.5%) males and 105 (17.5%) females were diagnosed SCD, among whom 58.0% of males and 34.3% of females lacked premortem medical records. A total of 1883 cases were collected from five external centers and 75.3% were males. The mean age at death was 50.4 years (standard deviation: 15.6 years). Three hundred seventy-one (26.2%) males and 70 (17.7%) females were diagnosed SCD. The detailed demographic and heart examination characteristics of the whole and each dataset were shown in Table 1 and Additional file 1: Table S1, respectively. The missingness rates of all datasets were less than 5% (Additional file 1: Fig. S1).

**Table 1:** Demographic and heart examination characteristics of study cohorts across six forensic centers

Characteristics^a^	Males in the training set		Females in the training set		Males in the test set		Females in the test set	
	SCD	Non-SCD	SCD	Non-SCD	SCD	Non-SCD	SCD	Non-SCD
*n*	581	1103	105	495	371	1046	70	396
Medical record available	244 (42.0)	652 (59.1)	69 (65.7)	352 (71.1)	194 (52.3)	672 (64.2)	44 (62.9)	262 (66.2)
Natural death (%)		637 (57.8)		350 (70.7)		418 (40.0)		183 (46.2)
Sudden death (%)		334 (30.3)		186 (37.6)		213 (20.4)		92 (23.2)
Sudden coronary artery death (%)	464 (79.9)		42 (40.0)		323 (87.1)		41 (58.6)	
Age (years)	48.8 ± 13.4	46.0 ± 14.7	50.7 ± 17.6	45.4 ± 16.0	51.5 ± 12.7	50.8 ± 15.7	51.9 ± 18.3	48.1 ± 17.2
Body height (cm)	166.9 ± 6.5	167.0 ± 7.1	155.7 ± 6.9	156.5 ± 6.1	168.8 ± 7.5	167.6 ± 7.6	156.6 ± 7.0	157.6 ± 7.0
Abdominal subcutaneous fat thickness (cm)^b^	2.16 ± 0.89	1.92 ± 1.10	2.71 ± 1.08	2.64 ± 1.13	2.70 ± 1.12	2.12 ± 1.06	3.24 ± 1.05	2.98 ± 1.25
Heart weight (g)	431 ± 106	385 ± 99	370 ± 98	328 ± 79	457 ± 121	378 ± 97	375 ± 100	315 ± 82
CAD (%)	425 (73.1)	185 (16.8)	38 (36.2)	46 (9.3)	312 (84.1)	314 (30.0)	40 (57.1)	52 (13.1)
MI (%)	224 (38.6)	70 (6.3)	26 (24.8)	6 (1.2)	226 (60.9)	88 (8.4)	32 (45.7)	22 (5.6)
Cardiomyopathy (%)	103 (17.7)	71 (6.4)	23 (21.9)	19 (3.8)	29 (7.8)	15 (1.4)	6 (8.6)	6 (1.5)
Left ventricular wall thickness (cm)	1.28 ± 0.20	1.26 ± 0.19	1.16 ± 0.16	1.14 ± 0.16	1.43 ± 0.27	1.34 ± 0.25	1.29 ± 0.23	1.22 ± 0.26
Right ventricular wall thickness (cm)	0.32 ± 0.08	0.31 ± 0.07	0.30 ± 0.09	0.29 ± 0.08	0.39 ± 0.13	0.36 ± 0.12	0.35 ± 0.13	0.32 ± 0.11
Circumference of tricuspid annulus (cm)	11.81 ± 0.77	11.62 ± 0.85	10.81 ± 1.36	10.85 ± 0.70	12.53 ± 1.09	12.13 ± 1.14	11.61 ± 0.98	11.23 ± 1.00
Circumference of pulmonary annulus (cm)	8.17 ± 0.72	7.89 ± 0.79	7.54 ± 0.69	7.34 ± 0.71	7.96 ± 0.96	7.73 ± 1.01	7.51 ± 1.07	7.12 ± 0.92
Circumference of mitral annulus (cm)	9.54 ± 0.87	9.32 ± 0.93	8.63 ± 1.43	8.67 ± 0.86	10.05 ± 1.06	9.64 ± 1.07	9.24 ± 1.13	8.83 ± 0.92
Circumference of aortic annulus (cm)	7.29 ± 0.62	7.13 ± 0.72	6.68 ± 0.72	6.57 ± 0.61	7.41 ± 0.83	7.26 ± 0.87	6.96 ± 0.91	6.53 ± 0.83

Abbreviation: *SCD* sudden cardiac death, *CAD* coronary artery disease, *MI* myocardial infarction

^a^Characteristics were summarized as frequency with percentage for nominal variable and mean with standard deviation for continuous variable with normal distribution

^b^Data of abdominal subcutaneous fat thickness were not available in China Medical University and Guizhou Medical University dataset

### Predictive value of the candidate variables for identifying SCD

Univariate and multivariate LR were applied in the SYSU dataset to explore the predictive value of candidate variables (Table 2). For demographic features, male, elder age and greater abdominal subcutaneous fat thickness were demonstrated to correlate with higher likelihood to suffer from SCD, with adjusted OR of 2.49 (95% CI: 1.98–3.16), 1.16 (per 10 years, 95% CI: 1.10–1.24) and 1.24 (per cm, 95% CI: 1.14–1.36). Furthermore, CAD, MI, and cardiomyopathy were demonstrated to be vital characterizations of SCD. Moreover, each 25% increase of stenosis in LAD, LCX and RCA was associated with a 2.34-fold (95% CI: 2.16–2.55), 2.80-fold (95% CI: 2.42–3.30) and 2.29-fold (95% CI: 2.08–2.53) elevated SCD risk, respectively. Furthermore, greater heart weight, LVWT, RVWT, cTA, cPA, cMA, and cAA were all found to be indicators for SCD (Table 2).

**Table 2:** Univariate and multivariate-adjusted associations between candidate variables and SCD according to the logistic regression model

Characteristics	Univariate analysis		Multivariate-adjusted analysis	
	OR (95% CI)	*P*	OR (95% CI)^a^	*P*
Sex (male vs female)	2.48 (1.97–3.15)	< 0.001	2.49 (1.98–3.16)	< 0.001
Age, per 10 years	1.16 (1.09–1.23)	< 0.001	1.16 (1.10–1.24)	< 0.001
Body height, per 10 cm	1.24 (1.11–1.38)	< 0.001	1.04 (0.90–1.19)	0.632
Abdominal subcutaneous fat thickness, per cm	1.08 (1.00–1.17)	0.052	1.24 (1.14–1.36)	< 0.001
CAD	12.3 (10.0–15.2)	< 0.001	12.4 (9.9–15.7)	< 0.001
MI	11.5 (8.70–15.2)	< 0.001	10.5 (7.90–14.1)	< 0.001
Stenosis degree of LAD, per 25%	2.31 (2.14–2.49)	< 0.001	2.34 (2.16–2.55)	< 0.001
Stenosis degree of LCX, per 25%	2.97 (2.56–3.49)	< 0.001	2.80 (2.42–3.30)	< 0.001
Stenosis degree of RCA, per 25%	2.37 (2.16–2.61)	< 0.001	2.29 (2.08–2.53)	< 0.001
Cardiomyopathy	3.77 (2.83–5.04)	< 0.001	3.58 (2.67–4.81)	< 0.001
Heart weight, per 100 g	1.67 (1.53–1.84)	< 0.001	1.53 (1.38–1.71)	< 0.001
Left ventricular wall thickness, per mm	1.13 (1.08–1.18)	< 0.001	1.03 (0.98–1.09)	0.237
Right ventricular wall thickness, per mm	1.33 (1.18–1.50)	< 0.001	1.17 (1.04–1.32)	0.009
Circumference of tricuspid annulus, per cm	1.43 (1.28–1.59)	< 0.001	1.17 (1.04–1.33)	0.013
Circumference of pulmonary annulus, per cm	1.79 (1.59–2.02)	< 0.001	1.54 (1.34–1.76)	< 0.001
Circumference of mitral annulus, per cm	1.36 (1.23–1.50)	< 0.001	1.16 (1.04–1.29)	0.009
Circumference of aortic annulus, per cm	1.58 (1.39–1.80)	< 0.001	1.24 (1.06–1.44)	0.006

Abbreviation: *OR* odds ratio, *CAD* coronary artery disease, *MI* myocardial infarction, *LAD* left anterior descending artery, *LCX* left circumflex artery, *RCA* right coronary artery

^a^The ORs were adjusted by age and sex for demographic variables and further adjusted by body height and abdominal subcutaneous fat thickness for cardiac morphological variables in multivariate logistics regression models

### Model development and selection of the optimal model

**Fig. 2:**
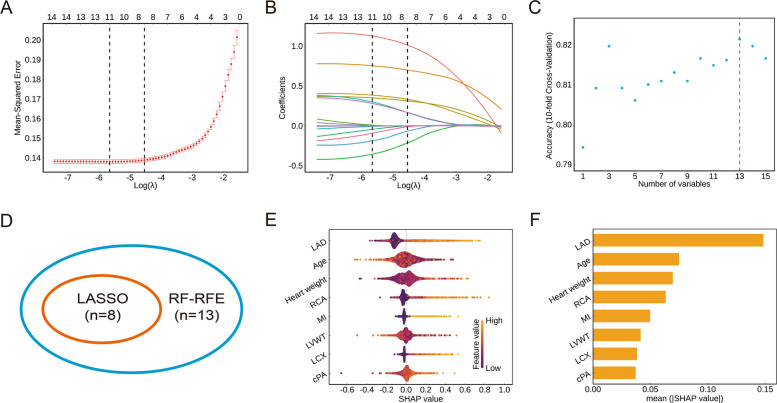
Feature selection for predicting SCD in the SYSU dataset (*n* = 2284). **A** Plot of mean squared error versus log (λ) in LASSO regression model with tenfold cross-validation. Dotted vertical lines are drawn at the optimal values by utilizing the minimum criteria (left line) and the 1 standard error criterion (right line, selected as the optimal penalty parameter in this study). **B** LASSO coefficient profiles. Curves represent coefficient profiles of features across regularization parameters (λ), with colors distinguishing individual variables. The optimal λ accounts for eight variables (including age, LAD, LCX, RCA, MI, heart weight, LVWT, and cPA) with eventually non-zero coefficients. **C** Scatter plot of accuracy versus number of variables selected in RF-RFE with tenfold cross-validation. The red dotted vertical line is drawn at the optimal number of variables (*n* = 13, including age, abdominal subcutaneous fat thickness, LAD, LCX, RCA, MI, heart weight, LVWT, RVWT, cAA, cPA, cMA, and cTA) that achieves the highest accuracy. **D** Venn diagram according to the relationship of variables selected by LASSO and RF-RFE method. **E** Positive and negative impact explanation of features for predicting SCD using SHAP values. The higher SHAP value of a feature is given, the higher probability of SCD the victim would be diagnosed. The yellow graduated color in feature value represents higher value. **F** The weights of variables importance. The higher the average of absolute SHAP value, the more important is the variable. Abbreviation: LASSO, least absolute shrinkage and selection operator; RF-RFE, recursive feature elimination based on random forest; LAD, left anterior descending artery; LCX, left circumflex artery; RCA, right coronary artery; MI, myocardial infarction; LVWT, left ventricle wall thickness; RVWT, right ventricle wall thickness; cTA, circumference of tricuspid annulus; cPA, circumference of pulmonary annulus; cMA, circumference of mitral annulus; cAA, circumference of aortic annulus; SHAP, Shapley Additive exPlanations

Both LASSO (Fig. 2A and B) and RF-RFE (Fig. 2C) approaches were applied for the feature selection in the SYSU dataset, after the standardization. Eight and thirteen variables were selected by these two methods respectively. The intersection including eight variables was applied as the final candidate (Fig. 2D), whose importance ranked as follow: LAD, age, heart weight, RCA, MI, LVWT, LCX, and cPA (Fig. 2E). Multicollinearity was not observed in the intersection (Additional file 1: Fig. S2).

**Fig. 3:**
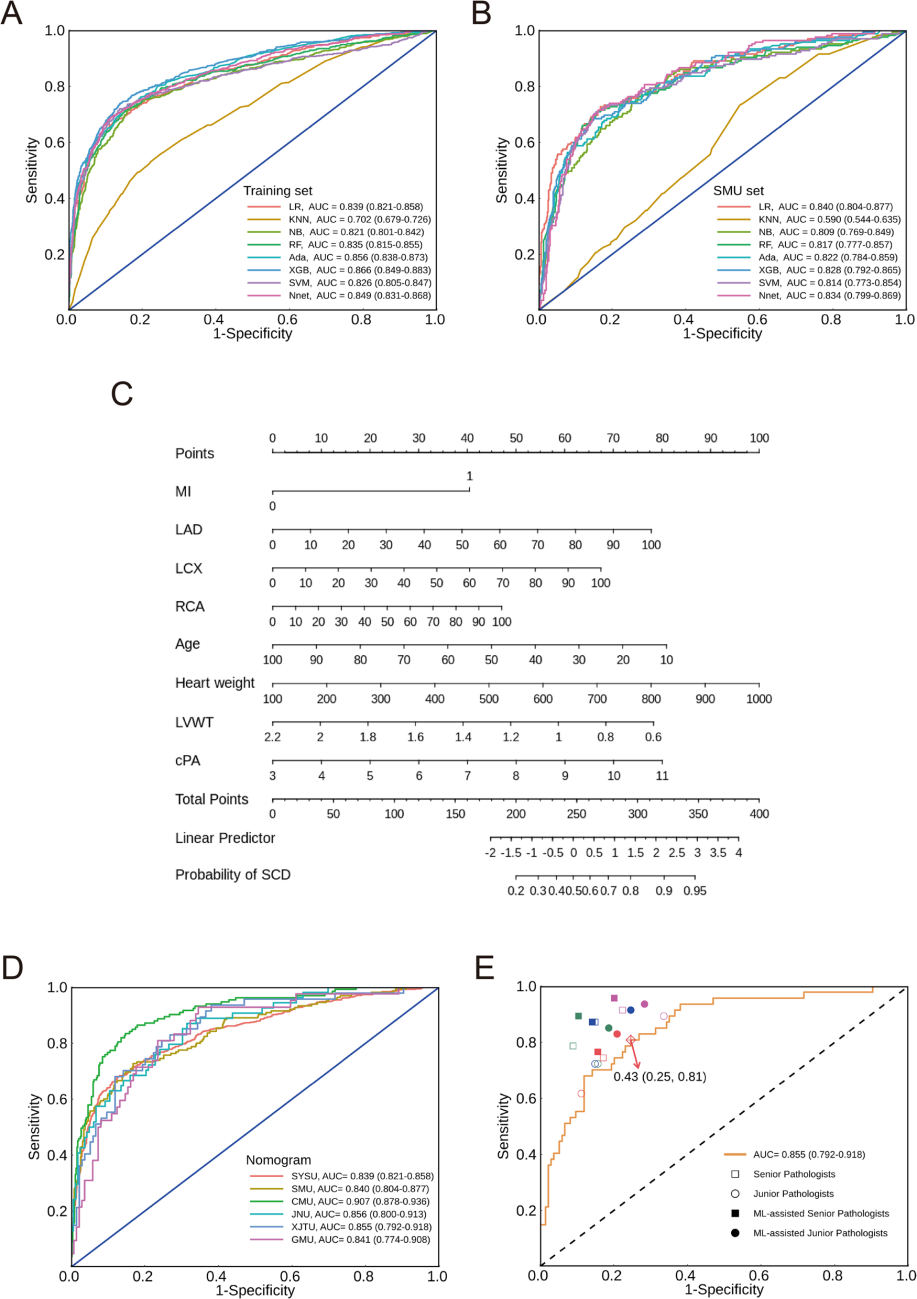
Model development, validation and human–machine fusion for the prediction of SCD. **A** ROC curves of eight ML models in the training set (*n* = 2284). **B** ROC curves of eight ML models in the SMU validation set (*n* = 741). **C** Nomogram derived from the LR model. **D** ROC curves of nomogram in the SYSU, SMU (*n* = 741), JNU (*n* = 216), CMU (*n* = 567), GMU (*n* = 178), and XJTU (*n* = 181) datasets. **E** Human–machine comparison and fusion experiment in the XJTU dataset (*n* = 181). The square and circular shape represent senior and junior pathologists, respectively. Each color corresponds to one pathologist participating in the experiment. The hollow and solid shape represent the performance of pathologists without and with the assistant of nomogram, respectively. Abbreviation: LR, logistics regression; KNN, *K*-nearest neighbor; NB, Naïve Bayes; RF, random forest; Ada, Adaboost; XGB, XGBoost; SVM, support vector machine; Nnet, single-hidden-layer neural network; MI, myocardial infarction; LAD, left anterior descending artery; LCX, left circumflex artery; RCA, right coronary artery; LVWT, left ventricle wall thickness; cPA, circumference of pulmonary annulus

Eight ML models were trained in the SYSU dataset, with AUC ranging from 0.702 (*K*-nearest neighbor) to 0.866 (XGBoost) (Fig. 3A). With the cut-off value determined by the maximum of Youden’s index, the accuracy of each model ranged from 0.692 (*K*-nearest neighbor) to 0.827 (Single-hidden-layer neural network). In the SMU dataset, the AUC ranged from 0.590 (*K*-nearest neighbor) to 0.840 (LR) and hence LR was elected as the final diagnostic model (Fig. 3B). The ROC curves of eight ML models in other four centers were shown in Additional file 1: Fig. S3 and statistical metrics in each dataset were summarized in Additional file 1: Table S2. A nomogram was developed (Fig. 3C) along with its corresponding web calculator.

### Model multicenter validation

The nomogram was validated in extra four forensic centers from different provinces in China and represented a robust performance, with the AUCs of 0.907 (95% CI: 0.878–0.936), 0.856 (95% CI: 0.800–0.913), 0.855 (95% CI: 0.792–0.918), and 0.841 (95% CI: 0.774–0.908) in the CMU, JNU, XJTU and GMU dataset, respectively (Fig. 3D). When the cutoff value of predicted probability was set as 0.36 based on the maximum of Youden’s index, the nomogram yielded 81.5% accuracy, 68.1% sensitivity, and 87.3% specificity in the SYSU dataset while the accuracy, sensitivity, and specificity ranged from 72.6% to 82.0%, from 66.7% to 86.5%, and from 70.9% to 83.8% in external centers, respectively (Additional file 1: Table S2). Furthermore, the statistical metrics corresponding to various cutoff values of the predicted probability of nomogram in each dataset were summarized in Additional file 1: Table S3 to enhance the understanding of model performance.

### Sensitivity and subgroup analysis

We firstly tested the impact of missingness on the efficiency of our model. The nomogram model was reconstructed in the training set with cases with missingness excluded. The AUCs of the reconstructed nomogram were 0.845 (95% CI: 0.826–0.864) in the training set and from 0.831 to 0.905 in external centers (Additional file 1: Fig. S4), which was similar to the results in imputed datasets.

We then tested the nomogram performance in population diagnosed as natural death and sudden death to simulate two forensic situations: cases with exogenous death causes excluded availably (based on witness information and routine forensic examinations) and further with death process witnessed, respectively. ROC curves represented that the nomogram achieved considerable performance in the prediction of SCD among cases inferred as natural death and sudden death, with an AUC of 0.818 (95% CI: 0.797–0.839) and 0.814 (95% CI: 0.791–0.838), respectively. The AUCs in external centers ranged from 0.802 (SMU dataset) to 0.874 (CMU dataset), and from 0.801 (SMU dataset) to 0.906 (CMU dataset), for the identification of SCD among natural death and sudden death, respectively (Additional file 1: Fig. S5).

Furthermore, we also explored the identification performance of SCD in different diseases. The nomogram yielded moderate AUC in cases with CAD (0.758, 95% CI: 0.733–0.783, Additional file 1: Fig. S6A) and cardiomyopathy (0.645, 95% CI: 0.580–0.710, Additional file 1: Fig. S6B).

### Human–machine fusion

We then launched a human–machine comparison and fusion experiment to test the application value of the nomogram in the XJTU dataset. In the human–machine comparison section, the AUCs of senior and junior pathologists ranged from 0.787 to 0.862 and from 0.753 to 0.787, respectively, which differed significantly (*P* = 0.02). The nomogram outperformed all of the four junior pathologists and three of four senior pathologists (Additional file 1: Table S4). With the assistance of nomogram, an increase of AUC and sensitivity was observed in both senior and junior pathologists (*P* = 0.004 and *P* = 0.01, respectively) (Fig. 3E). In details, one of the four senior pathologists and three of the four junior pathologists achieved a significant improvement of sensitivity, and an upgrade of specificity was also observed in a half of junior pathologists. The detailed statistical tests were summarized in Additional file 1: Table S4.

### Clinical translation

**Fig. 4:**
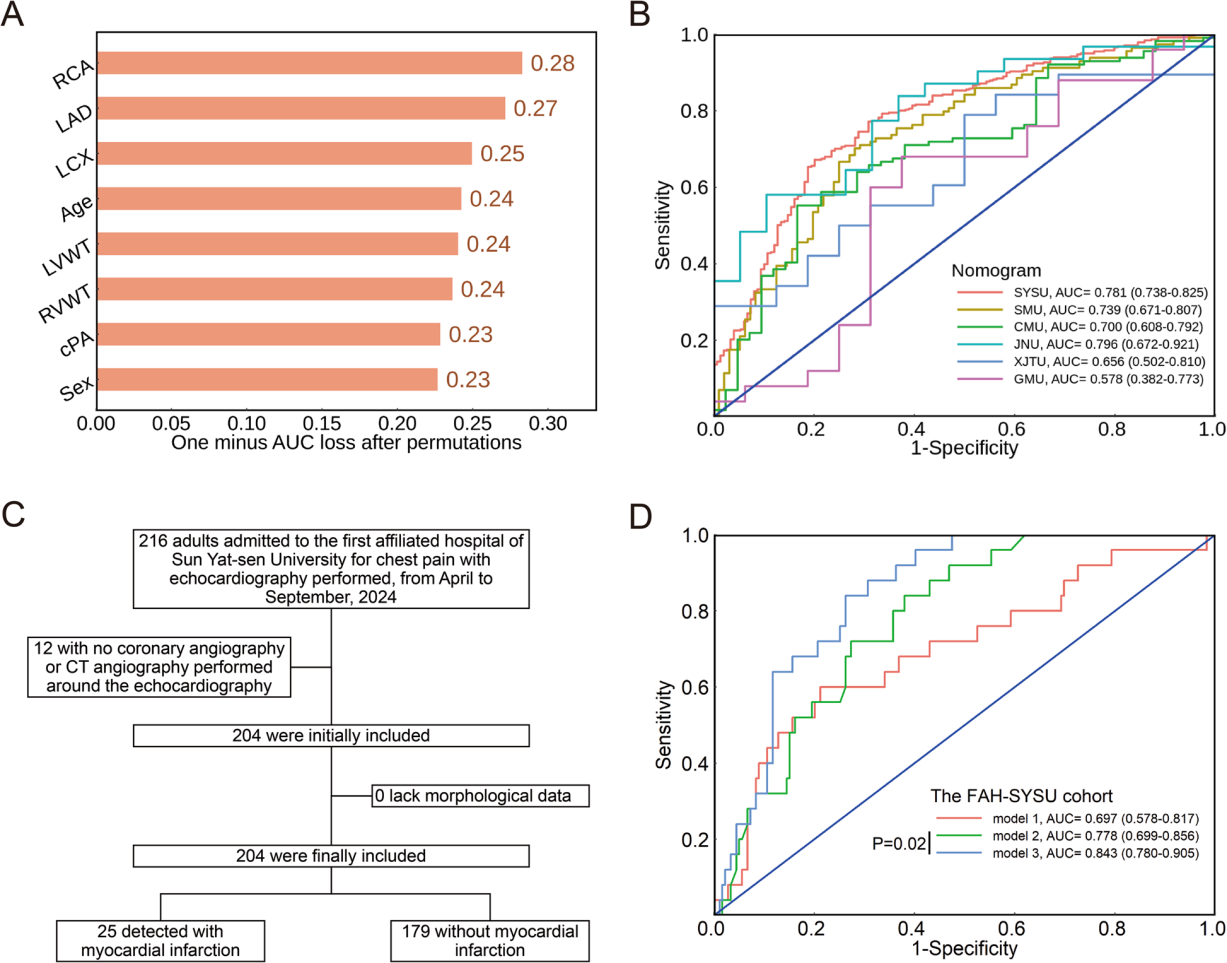
Interdisciplinary translation from the forensic diagnosis of sudden coronary artery death among individuals with coronary artery disease to the clinical prediction of MI among patients with chest pain. **A** Single-permutation-based variable-importance measures in the LR model for variables in the interaction of LASSO and RF-RFE method using 1-AUC as the loss function. The larger the change in the performance, the more important is the variable. **B** ROC curves of the LR model for the diagnosis of sudden coronary artery death in the SYSU (*n* = 530), SMU (*n* = 210), JNU (*n* = 50), CMU (*n* = 156), GMU (*n* = 41), and XJTU (*n* = 54) datasets. **C** The flow chart of the clinical cohort from the First Affiliated Hospital of Sun Yat-sen University. **D** ROC curves of the LR model for the prediction of MI in the clinical cohort (*n* = 204). Model 1 includes sex, age and echocardiography-variables selected from forensic setting (thickness of left ventricular posterior wall and right ventricular anterior wall, and diameter of pulmonary valve). Model 2 acts as the reference model, which only includes the stenosis degree of LAD, LCX and RCA. Model 3 includes all the converted variables in the interaction of LASSO and RF-RFE methods, including stenosis degree of LAD, LCX and RCA, sex, age, thickness of left ventricular posterior wall and right ventricular anterior wall, and diameter of pulmonary valve. The comparison between model 2 and 3 was conducted using a one-tailed DeLong’s test. Abbreviation: LVWT, left ventricle wall thickness; RVWT, right ventricle wall thickness; cPA, circumference of pulmonary annulus; LAD, stenosis degree of left anterior descending artery; LCX, stenosis degree of left circumflex artery; RCA, stenosis degree of right coronary artery; LASSO, least absolute shrinkage and selection operator; RF-RFE, recursive feature elimination based on random forest

In the SYSU dataset (*n* = 530), both LASSO and RF-RFE methods were applied for the feature selection, with eight and eleven variables selected, respectively (Additional file 1: Fig. S7A–C). The interaction, which included RCA, LAD, LCX, age, LVWT, RVWT, cPA, and sex (ranks based on the feature importance, Additional file 1: Fig. S7D and Fig. 4A), achieved a moderate performance in the prediction of sudden coronary artery death using LR model, with an AUC of 0.781 (95% CI: 0.738–0.825) in the SYSU dataset. AUCs of the LR model in other forensic centers ranged from 0.578 to 0.796 (Fig. 4B).

A total of 204 patients with chest pain were enrolled in this study and 189 (92.6%) received coronary angiography. Twenty-five individuals (12.3%) were detected with MI (Fig. 4C). Among the variables selected in the forensic dataset and converted to be echocardiography-available (Additional file 1: Fig. S8), left ventricular posterior wall thickness was found to be higher in patients with MI (10.32 ± 1.52 mm vs 9.64 ± 1.34 mm, *P* = 0.02, Additional file 1: Table S5). The LR model including age, sex and echocardiography-measurable left ventricular posterior wall thickness, right ventricular anterior wall thickness, and pulmonary valve diameter, yielded an AUC of 0.697 (95% CI: 0.578–0.817) in MI identification. The AUC significantly increased with the addition of morphological variables, compared to the sole combination of stenosis degree of three main coronary branches (0.843 [95% CI: 0.780–0.905] vs. 0.778 [95% CI: 0.669–0.856], *P* = 0.02, Fig. 4D).

## Discussion

In this study, we conducted a large-scale autopsy dataset and carefully constructed a standard autopsy-based ML model for the quantitative diagnosis of SCD. The well-tuned model yielded considerable performance in the training set, achieved a satisfactory generalizability across forensic centers, and was verified to be effective in human–machine fusion experiment. The forensic autopsy-to-clinical echocardiography transformation also validated the potential of the morphology-based model in MI identification.

In the absence of medical records and witness accounts, out-of-hospital diagnosis of SCD remains particularly challenging in forensic investigations. There was usually divergence on the final diagnosis between different pathologists due to the lack of a unified evaluation standard for heart lesions. To achieve an accurate diagnosis of SCD, we developed an autopsy-based ML framework that quantifies cardiac lesion severity to improve SCD diagnosis in resource-limited forensic settings. A LR-nomogram including eight variables performed robustly in the training set and five external centers distributed in four representative provinces of China. Due to the only requirement of routine autopsy data, the well-validated model could assist in indicating the probability of dying of SCD in forensic practice settings where external evidence is limited. Subgroup analyses in natural death and sudden death further demonstrated the model’s robust performance across diverse forensic scenarios. The nomogram achieved an accuracy comparable to senior pathologists (> 10 years’ experience) and significantly improved the accuracy and sensitivity among both senior and junior practitioners, highlighting its value as a decision-support tool.

In a full autopsy with sequential approach, SCD is diagnosed with natural cardiac lesions observed and extra-cardiac fatal causes excluded. Many cardiovascular lesions can account for either electric or mechanical SCD by influencing coronary arteries, myocardium, heart valves, the conduction system, and so on [3], which are also the focus of the macroscopic and histologic examinations. Besides typical fatal lesions, several anatomical features are also declared to be associated with SCD, including abdominal fatness, heart weight, LVWT and circumference of cardiac valves [28–32], which can provide extra information to convince the diagnosis, especially when the typically fatal lesions are absent. The core problem is how to estimate the diagnostic contribution and then the weight of these single variables in a case and hence a framework that not only concentrates on decisive lesions, but also considers other coexisting anatomical features is required.

Based on more than four thousand rigorously diagnosed cases, the well-developed and validated system clarified the predictive potential of morphological and histopathological features and may assist in the diagnosis of SCD. In application, it gives a quantitative probability of SCD in a case so that pathologists can conclude a diagnosis by comparing the probability with cut-off values, with desired accuracy, sensitivity, specificity, negative predictive value and positive predictive value. The nomogram specializing at high sensitivity may be a helpful tool for reducing false dismissal rate, as suggested by the human–machine fusion experiment. Besides, the system also has advantages in terms of interpretability and simplicity. The nomogram gives a validated weight for typical histopathological findings, demographic variables and macro-morphological features and SHAP methods provide the feature importance ranks and the contributions of each variables in the diagnosis so that pathologists can understand the diagnostic value of each feature better and generate a diagnosis in the complex setting of comorbidities, such as the simultaneous narrowing of several branches of coronary artery, with no other fatal histopathological etiologies observed. We also extended the LR-nomogram model to a web server so that the pathologists can conveniently receive timely feedback about the probability of diagnosing SCD from the web calculator.

To increase the generality in out-of-hospital diagnosis and extend the clinical significance, our model concentrating on cardiac lesions and only recruited routine variables. Meanwhile, as our model only recruited routine autopsy findings, a few autopsy-negative SCD would be classified incorrectly, which requires advanced and specific methods including premortem monitoring, genetic tests, specific histopathological examinations, and so on [33, 34]. Therefore, the cardiac morphology-based nomogram should be considered as an assistant tool in forensic practice and a final diagnosis should be made under a full analysis of autopsy findings, differential information, and other necessary examinations.

Beyond postmortem diagnosis, SCD prevention is a critical unmet need in global cardiovascular medicine [1]. In recent decades, left and right ventricular hypertrophy have been demonstrated to correlate with a higher risk of cardiovascular death [17, 19]. However, clinically applicable risk-predicting models integrating cardiac morphological features remain underdeveloped, despite their potential utility [35, 36]. As SCD is a terminal of cardiac diseases, our model constructed utilizing forensic data considering heart morphological variables during autopsy and with SCD as the outcome may provide insights into heart burden and could potentially contribute to identifying patients at higher risk of SCD. To validate the clinical value of our model, we prospectively conducted a pilot cohort to apply the transformed model from forensic systematic autopsy to clinical echocardiography in the identification of MI, which accounts for the majority of SCD. Patients with MI were found to have a greater left ventricular posterior wall thickness and the combination of morphological variables converted from anatomically to echocardiography measurable, yielded a moderate performance in MI identification. As a noninvasive, low-cost, and easily available bedside imaging tool, echocardiography is demonstrated to have prognostic role in the early risk identification after acute MI [35]. These preliminary findings suggest echocardiography-derived morphological parameters help in the rapid and quantitative evaluation of the heart burden and may enhance MI risk stratification, particularly when combined with traditional indicators (e.g., blood pressure, diabetes comorbidity, dyslipidemia, and family history) [37, 38]. Meanwhile, the initial success in clinical echocardiography cohort also raises the potential of a virtual autopsy by transforming the morphology-based model to be imaging technology-available in the forensic diagnosis of SCD, which exempts from performing an actual autopsy.

Several limitations merit consideration. First, this diagnostic model only acted as a heart lesion evaluation system to increase the generality in out-of-hospital diagnosis and extend its clinical significance. Occurrences of competitive injuries or diseases would account for a few false positive misdiagnosing and lower the model’s specificity. Second, the diagnostic capability of the nomogram among individuals dying of scarce cardiac lesions (such as heart inflammation and conduction system lesions) is not fully validated and hence a specific study concentrating on these death causes is warranted. Furthermore, as our study only recruited cases from Chinese forensic centers, the morphological features of other ethnicities should be further figured out and a further validation with necessary calibration of our model in other ethnicities is warranted before its international application. Finally, the limited sample size of the clinical validation cohort reduced statistical power for detecting associations with infrequent clinical features, necessitating larger-scale studies. A large-scale cohort study has been scheduled for the validation of predictive value of these echocardiography-available heart morphological features.

## Conclusions

This multicenter study developed and validated an autopsy-based ML system for cardiac lesion evaluation, demonstrating robust performance for SCD diagnosis across diverse forensic scenarios, which is comparable to senior pathologists. Human–machine fusion experiment further validated its assistant potential. The transformed morphological-based model also presented moderate performance in clinical identification of MI in a pilot prospective non-randomized cohort.

## Supplementary Information

Additional file 1. Supplementary methods. Table S1. Demographic and heart examination characteristics of study cohorts in five external centers. Table S2. Statistical metrics for each machine learning model in the six datasets. Table S3. Statistical metrics of nomogram corresponding to various cutoff value in the six datasets. Table S4. Statistical metrics according to the human-machine comparison and fusion experiment. Table S5. Characteristics of patients in clinical cohort. Fig. S1. Missingness map of datasets of six forensic centers. Fig. S2. The correlation heatmap of variables in the interaction of LASSO and RF-RFE method. Fig. S3. ROC curves of eight ML models in four external datasets. Fig. S4. Sensitivity analysis about the missingness during the model construction. Fig. S5. Subgroup analyses of the nomogram in natural death and sudden death mode. Fig. S6. Subgroup analyses of the nomogram in different diseases. Fig. S7. Feature selection for the prediction of sudden coronary artery death among individuals with coronary artery disease in forensic setting. Fig. S8. The two-dimensional echocardiography-available measurement of heart morphological features

## Data Availability

The datasets generated for this study are available on reasonable request from the corresponding authors.

## Abbreviations

SCD: Sudden cardiac death
CAD: Coronary artery disease
ML: Machine learning
MI: Myocardial infarction
SYSU: Sun Yat-sen University
SMU: Southern Medical University
JNU: Jinan University
CMU: China Medical University
GMU: Guizhou Medical University
XJTU: Xi’an Jiaotong University
LVWT: Left ventricle wall thickness
RVWT: Right ventricle wall thickness
cTA: Circumference of tricuspid annulus
cPA: Circumference of pulmonary annulus
cMA: Circumference of mitral annulus
cAA: Circumference of aortic annulus
LAD: Left anterior descending artery
LCX: Left circumflex artery
RCA: Right coronary artery
LASSO: Least absolute shrinkage and selection operator
RF-RFE: Recursive feature elimination based on random forest
SHAP: Shapley Additive exPlanations
XGBoost: Extreme gradient boosting trees
LR: Logistic regression
AUC: Area under receiver-operating characteristic curve
ROC: Receiver-operating characteristic
