# Supplementary material for: An autopsy-based cardiac lesion evaluation system facilitates quantitative diagnosis of sudden cardiac death: development and multicenter validation of a machine learning model
Source: BMC Med. 2025 Nov 26;23:712. doi: 10.1186/s12916-025-04529-6 (PMC12751744; doi:10.1186/s12916-025-04529-6)
Supplement: Supplementary file 1 — Additional file 1. Supplementary methods. Table S1. Demographic and heart examination characteristics of study cohorts in five external centers. Table S2. Statistical metrics for each machine learning model in the six datasets. Table S3. Statistical metrics of nomogram corresponding to various cutoff value in the six datasets. Table S4. Statistical metrics according to the human-machine comparison and fusion experiment. Table S5. Characteristics of patients in clinical cohort. Fig. S1. Missingness map of datasets of six forensic centers. Fig. S2. The correlation heatmap of variables in the interaction of LASSO and RF-RFE method. Fig. S3. ROC curves of eight ML models in four external datasets. Fig. S4. Sensitivity analysis about the missingness during the model construction. Fig. S5. Subgroup analyses of the nomogram in natural death and sudden death mode. Fig. S6. Subgroup analyses of the nomogram in different diseases. Fig. S7. Feature selection for the prediction of sudden coronary artery death among individuals with coronary artery disease in forensic setting. Fig. S8. The two-dimensional echocardiography-available measurement of heart morphological features [file 12916_2025_4529_MOESM1_ESM.pdf]

An autopsy-based cardiac lesion evaluation system facilitates quantitative diagnosis of sudden cardiac death: development and multicenter validation of a machine learning model

Additional file 1

List of contents

Supplementary methods 2

Table S1. Demographic and heart examination characteristics of study cohorts in five external centers 3

Table S2. Statistical metrics for each machine learning model in the six datasets 4

Table S3. Statistical metrics of nomogram corresponding to various cutoff value in the six datasets 7

Table S4. Statistical metrics according to the human-machine comparison and fusion experiment 9

Table S5. Characteristics of patients in clinical cohort 10

Figure S1. Missingness map of datasets of six forensic centers 11

Figure S2. The correlation heatmap of variables in the interaction of LASSO and RF-RFE method 12

Figure S3. ROC curves of eight ML models in four external datasets 13

Figure S4. Sensitivity analysis about the missingness during the model construction 14

Figure S5. Subgroup analyses of the nomogram in natural death and sudden death mode 15

Figure S6. Subgroup analyses of the nomogram in different diseases 16

Figure S7. Feature selection for the prediction of sudden coronary artery death among individuals with coronary artery disease in forensic setting 17

Figure S8. The two-dimensional echocardiography-available measurement of heart morphological features 18

Supplementary methods

The heart was separated from major vessels and heart weight was measured after removing the intracardiac blood. Left ventricular posterior wall and right ventricular anterior wall thickness were measured at mid-ventricular level (about 2 cm inferior to the coronary sulcus). After cutting the heart along with cardiac inflow-outflow axes, the circumferences of tricuspid, pulmonary, mitral and aortic valve annuli were recorded. Serial cross sections of left anterior descending branch, left circumflex branch and right coronary artery were obtained in situ at 2–3 mm intervals. Segments exhibiting maximal luminal narrowing of each artery were selected for histopathological processing: fixed in 10% neutral buffered formalin, paraffin-embedded, sectioned at 4-μm thickness, and stained with hematoxylin-eosin. Whole-slide imaging was performed at 20× optical magnification, with luminal stenosis quantification executed through a standardized protocol: Stenosis degree = (1- Residual lumen area/inner elastic membrane enclosed area) *100% implemented in ImageJ software. Myocardial infarction was detected by experienced pathologists according to specific characteristics (coagulation, contraction band necrosis, or focal myocardial scar).

Table S1. Demographic and heart examination characteristics of study cohorts in five external centers

Characteristics[†]	SMU	CMU	JNU	XJTU	GMU
n	741	567	216	181	178
Natural death (%)	431 (58.2)	258 (45.5)	131 (60.6)	100 (55.2)	121 (68.0)
Sudden death (%)	302 (40.8)	184 (32.5)	107 (49.5)	84 (46.4)	69 (38.8)
SCD (%)	165 (22.3)	133 (23.5)	54 (25.0)	47 (26.0)	43 (24.2)
Sudden coronary artery death (%)	128 (17.3)	115 (20.3)	44 (20.4)	42 (23.2)	35 (19.7)
Male (%)	550 (74.2)	444 (78.3)	178 (82.4)	123 (68.0)	122 (68.5)
Age (year)	49.6±16.2	53.8±14.6	47.5±13.9	48.2±16.0	48.7±16.4
Body height (cm)	163.6±8.5	169.2±8.4	164.4±8.0	165.0±8.3	161.6±7.6
Abdominal subcutaneous fat thickness (cm)[‡]	2.45 ±1.19		2.21 ±1.07	2.81 ±1.22	
Heart weight (g)	372±111	397±111	387±99	354±106	378±106
CAD (%)	298 (40.2)	218 (38.4)	79 (36.6)	72 (39.8)	51 (28.7)
MI (%)	129 (17.4)	120 (21.2)	39 (18.1)	43 (23.8)	37 (20.8)
Cardiomyopathy (%)	22 (3.0)	12 (2.1)	16 (7.4)	4 (2.2)	2 (1.1)
Left ventricular wall thickness (cm)	1.35±0.23	1.44±0.27	1.23±0.22	1.11±0.27	1.25±0.23
Right ventricular wall thickness (cm)	0.36±0.11	0.42±0.12	0.32±0.07	0.26±0.12	0.30±0.12
Circumference of tricuspid annulus (cm)	11.89±1.08	12.33±1.17	11.54±1.13	12.12±1.52	11.82±0.95
Circumference of pulmonary annulus (cm)	7.36±1.01	8.00±0.93	7.66±0.88	8.02±1.17	7.24±0.88
Circumference of mitral annulus (cm)	9.28±1.04	9.85±1.14	9.37±0.99	10.02±1.33	9.31±0.90
Circumference of aortic annulus (cm)	6.96±0.84	7.51±0.90	6.93±0.77	7.27±0.99	6.67±0.83

† Characteristics were summarized as frequency with percentage for nominal variable and mean with standard deviation for continuous variable with normal distribution.

‡ Data of abdominal subcutaneous fat thickness were not available in CMU and GMU dataset.

Abbreviation: SMU, Southern Medical University; CMU, China Medical University; JNU, Jinan University; XJTU, Xi'an Jiaotong University; GMU, Guizhou Medical University; SCD, sudden cardiac death; CAD, coronary artery disease; MI, myocardial infarction.

Table S2. Statistical metrics for each machine learning model in the six datasets

Model	Cut-off value†	Center	Accuracy (95%CI)	Sensitivity	Specificity	Positive predictive value	Negative predictive value	F1 score	Kappa
Logistic regression	0.360	SYSU	0.815 (0.799-0.831)	0.681	0.873	0.697	0.864	0.689	0.557
		SMU	0.726 (0.692-0.758)	0.764	0.715	0.434	0.914	0.554	0.377
		CMU	0.820 (0.786-0.851)	0.865	0.806	0.578	0.951	0.693	0.573
		JNU	0.773 (0.711-0.827)	0.685	0.802	0.536	0.884	0.602	0.446
		XJTU	0.740 (0.670-0.803)	0.830	0.709	0.500	0.922	0.624	0.444
		GMU	0.798 (0.731-0.854)	0.667	0.838	0.560	0.891	0.609	0.474
K-nearest neighbor	0.202	SYSU	0.692 (0.672-0.711)	0.560	0.748	0.489	0.798	0.522	0.296
		SMU	0.630 (0.594-0.665)	0.358	0.708	0.260	0.794	0.301	0.058
		CMU	0.686 (0.646-0.724)	0.519	0.737	0.377	0.833	0.437	0.227
		JNU	0.653 (0.585-0.716)	0.519	0.698	0.364	0.813	0.427	0.189
		XJTU	0.619 (0.544-0.690)	0.404	0.694	0.317	0.769	0.355	0.090
		GMU	0.764 (0.695-0.824)	0.548	0.831	0.500	0.856	0.523	0.366
Gaussian Naïve Bayes	0.052	SYSU	0.796 (0.778-0.812)	0.724	0.826	0.641	0.875	0.680	0.531
		SMU	0.645 (0.609-0.680)	0.873	0.580	0.373	0.941	0.523	0.306
		CMU	0.728 (0.690-0.765)	0.940	0.664	0.461	0.973	0.619	0.444
		JNU	0.759 (0.697-0.815)	0.833	0.735	0.511	0.930	0.634	0.469
		XJTU	0.685 (0.612-0.752)	0.851	0.627	0.444	0.923	0.584	0.368
		GMU	0.787 (0.719-0.844)	0.786	0.787	0.532	0.922	0.635	0.492
Random forest	0.293	SYSU	0.806 (0.789-0.822)	0.717	0.844	0.663	0.874	0.689	0.548
		SMU	0.638 (0.603-0.673)	0.855	0.576	0.366	0.933	0.513	0.292
		CMU	0.746 (0.708-0.781)	0.902	0.698	0.478	0.959	0.625	0.459
		JNU	0.745 (0.682-0.802)	0.796	0.728	0.494	0.915	0.610	0.436
		XJTU	0.729 (0.658-0.793)	0.851	0.687	0.488	0.929	0.620	0.433
		GMU	0.770 (0.701-0.829)	0.738	0.779	0.508	0.906	0.602	0.448

AdaBoost	0.376	SYSU	0.813 (0.796-0.828)	0.717	0.854	0.678	0.875	0.697	0.561
		SMU	0.669 (0.634-0.703)	0.830	0.623	0.387	0.928	0.528	0.322
		CMU	0.781 (0.745-0.815)	0.887	0.749	0.520	0.956	0.656	0.511
		JNU	0.759 (0.697-0.815)	0.741	0.765	0.513	0.899	0.606	0.441
		XJTU	0.718 (0.647-0.782)	0.787	0.694	0.474	0.903	0.592	0.396
		GMU	0.775 (0.707-0.834)	0.667	0.809	0.519	0.887	0.583	0.433
XGBoost	0.284	SYSU	0.824 (0.808-0.839)	0.739	0.860	0.695	0.885	0.716	0.589
		SMU	0.684 (0.649-0.718)	0.812	0.648	0.398	0.923	0.534	0.335
		CMU	0.797 (0.762-0.830)	0.887	0.770	0.541	0.957	0.672	0.538
		JNU	0.787 (0.726-0.840)	0.833	0.772	0.549	0.933	0.662	0.516
		XJTU	0.757 (0.688-0.817)	0.894	0.709	0.519	0.950	0.656	0.488
		GMU	0.792 (0.725-0.849)	0.690	0.824	0.547	0.896	0.611	0.471
Support vector machine	0.189	SYSU	0.818 (0.802-0.834)	0.720	0.860	0.689	0.877	0.704	0.573
		SMU	0.637 (0.601-0.672)	0.848	0.576	0.365	0.930	0.510	0.288
		CMU	0.755 (0.717-0.790)	0.880	0.717	0.487	0.951	0.627	0.466
		JNU	0.782 (0.721-0.836)	0.796	0.778	0.544	0.920	0.647	0.497
		XJTU	0.735 (0.664-0.798)	0.872	0.687	0.494	0.939	0.631	0.448
		GMU	0.792 (0.725-0.849)	0.762	0.801	0.542	0.916	0.634	0.494
Single-hidden-layer neural network	0.345	SYSU	0.827 (0.811-0.842)	0.690	0.886	0.722	0.869	0.705	0.583
		SMU	0.726 (0.692-0.758)	0.806	0.703	0.438	0.927	0.567	0.392
		CMU	0.824 (0.790-0.854)	0.880	0.806	0.582	0.956	0.701	0.583
		JNU	0.792 (0.731-0.844)	0.759	0.802	0.562	0.909	0.646	0.503
		XJTU	0.773 (0.706-0.832)	0.851	0.746	0.541	0.935	0.661	0.503
		GMU	0.803 (0.737-0.859)	0.690	0.838	0.569	0.898	0.624	0.492

† The cut-off value was determined by the maximum of Youden's index in the training set (the SYSU dataset).

Abbreviation: SYSU, Sun Yat-sen University; SMU, Southern Medical University; CMU, China Medical University; JNU, Jinan University; XJTU, Xi'an Jiaotong University; GMU, Guizhou Medical University.

Table S3. Statistical metrics of nomogram corresponding to various cutoff value in the six datasets

Center	Cut-off value	Accuracy	Sensitivity	Specificity	Youden's index	Positive predictive value	Negative predictive value	*F*1 score	Kappa
SYSU	0.2	0.749	0.777	0.737	0.514	0.559	0.885	0.650	0.463
	0.3	0.794	0.710	0.830	0.540	0.642	0.870	0.675	0.525
	0.4	0.820	0.640	0.898	0.538	0.729	0.853	0.682	0.557
	0.5	0.824	0.567	0.934	0.501	0.787	0.834	0.659	0.545
	0.6	0.814	0.487	0.955	0.442	0.823	0.813	0.612	0.500
	0.7	0.801	0.407	0.970	0.377	0.853	0.792	0.551	0.443
	0.8	0.780	0.309	0.982	0.291	0.880	0.768	0.457	0.357
	0.9	0.749	0.179	0.994	0.173	0.925	0.738	0.300	0.225
Whole test set†	0.2	0.700	0.889	0.643	0.532	0.432	0.950	0.582	0.389
	0.3	0.748	0.810	0.730	0.540	0.478	0.926	0.601	0.434
	0.4	0.783	0.773	0.786	0.559	0.525	0.919	0.626	0.481
	0.5	0.813	0.723	0.840	0.563	0.581	0.909	0.644	0.520
	0.6	0.834	0.685	0.880	0.565	0.636	0.901	0.659	0.550
	0.7	0.839	0.594	0.914	0.508	0.679	0.880	0.634	0.531
	0.8	0.844	0.515	0.945	0.460	0.742	0.864	0.608	0.515
	0.9	0.837	0.390	0.974	0.364	0.823	0.839	0.529	0.446
SMU	0.2	0.648	0.885	0.580	0.465	0.376	0.946	0.528	0.314
	0.3	0.700	0.782	0.677	0.459	0.410	0.915	0.537	0.347
	0.4	0.746	0.758	0.743	0.501	0.458	0.915	0.571	0.406
	0.5	0.796	0.733	0.814	0.547	0.531	0.914	0.616	0.482
	0.6	0.818	0.691	0.854	0.545	0.576	0.906	0.628	0.509
	0.7	0.833	0.618	0.894	0.512	0.626	0.891	0.622	0.515
	0.8	0.853	0.570	0.934	0.504	0.712	0.883	0.633	0.542
	0.9	0.845	0.394	0.974	0.368	0.813	0.849	0.531	0.451
CMU	0.2	0.748	0.910	0.698	0.608	0.480	0.962	0.629	0.464
	0.3	0.799	0.880	0.774	0.654	0.544	0.955	0.672	0.539
	0.4	0.832	0.857	0.825	0.682	0.600	0.950	0.706	0.594
	0.5	0.850	0.805	0.864	0.669	0.645	0.935	0.716	0.616
	0.6	0.866	0.774	0.894	0.668	0.691	0.928	0.730	0.642
	0.7	0.869	0.669	0.931	0.600	0.748	0.902	0.706	0.623
	0.8	0.859	0.549	0.954	0.503	0.785	0.873	0.646	0.561
	0.9	0.854	0.436	0.982	0.418	0.879	0.850	0.583	0.506
JNU	0.2	0.731	0.870	0.685	0.555	0.480	0.941	0.618	0.437
	0.3	0.759	0.759	0.759	0.518	0.513	0.904	0.612	0.447
	0.4	0.801	0.685	0.840	0.525	0.587	0.889	0.632	0.497
	0.5	0.806	0.630	0.864	0.494	0.607	0.875	0.618	0.488
	0.6	0.838	0.574	0.926	0.500	0.721	0.867	0.639	0.536
	0.7	0.833	0.481	0.951	0.432	0.765	0.846	0.591	0.493
	0.8	0.838	0.426	0.975	0.401	0.852	0.836	0.568	0.481
	0.9	0.819	0.333	0.981	0.314	0.857	0.815	0.480	0.395

XJTU	0.2	0.657	0.936	0.560	0.496	0.427	0.962	0.587	0.358
	0.3	0.724	0.851	0.679	0.530	0.482	0.929	0.615	0.425
	0.4	0.751	0.809	0.731	0.540	0.514	0.916	0.628	0.455
	0.5	0.768	0.745	0.776	0.521	0.538	0.897	0.625	0.463
	0.6	0.807	0.702	0.843	0.545	0.611	0.890	0.653	0.520
	0.7	0.812	0.617	0.881	0.498	0.644	0.868	0.630	0.505
	0.8	0.812	0.511	0.918	0.429	0.686	0.842	0.585	0.467
	0.9	0.818	0.468	0.940	0.408	0.733	0.834	0.571	0.463
GMU	0.2	0.775	0.810	0.765	0.575	0.515	0.929	0.630	0.480
	0.3	0.798	0.714	0.824	0.538	0.556	0.903	0.625	0.489
	0.4	0.792	0.643	0.838	0.481	0.551	0.884	0.593	0.455
	0.5	0.820	0.524	0.912	0.436	0.647	0.861	0.579	0.466
	0.6	0.826	0.500	0.926	0.426	0.677	0.857	0.575	0.469
	0.7	0.803	0.381	0.934	0.315	0.640	0.830	0.478	0.366
	0.8	0.803	0.310	0.956	0.266	0.684	0.818	0.426	0.327
	0.9	0.798	0.214	0.978	0.192	0.750	0.801	0.333	0.255

† The whole test set comprises five external validation cohorts (SMU, CMU, JNU, XJTU and GMU). Abbreviation: SYSU, Sun Yat-sen University; SMU, Southern Medical University; CMU, China Medical University; JNU, Jinan University; XJTU, Xi'an Jiaotong University; GMU, Guizhou Medical University.

Table S4. Statistical metrics according to the human-machine comparison and fusion experiment

	Human-machine comparison†							Human-machine fusion‡						
	AUC	$P^{\#}$	Accuracy	Sensitivity	$P^{\S}$	Specificity	$P^{\S}$	AUC	$P^{\#}$	Accuracy	Sensitivity	$P^{\S}$	Specificity	$P^{\S}$
Nom	0.855		0.729	0.830		0.694								
SP 1	0.849	0.86	0.878	0.787	0.53	0.91	<0.0001*	0.895	0.056	0.895	0.894	0.025*	0.896	0.317
SP 2	0.846	0.71	0.812	0.915	0.10	0.776	0.048*	0.878	0.045*	0.840	0.957	0.157	0.799	0.083
SP 3	0.862	0.86	0.856	0.872	0.56	Mn	0.0003*	0.865	0.317	0.862	0.872	1.000	0.858	0.317
SP 4	0.787	0.09	0.807	0.745	0.25	0.828	0.002*	0.805	0.127	0.823	0.766	0.317	0.843	0.157
JP 1	0.783	0.050*	0.812	0.723	0.13	0.843	0.0004*	0.832	0.123	0.823	0.851	0.034*	0.813	0.248
JP 2	0.779	0.019*	0.724	0.894	0.26	0.664	0.48	0.826	0.018*	0.773	0.936	0.157	0.716	0.052
JP 3	0.787	0.072	0.818	0.723	0.17	0.851	0.0002*	0.834	0.142	0.796	0.915	0.003*	0.754	0.001*
JP 4	0.753	0.007*	0.818	0.617	0.004*	0.888	<0.0001*	0.810	0.089	0.801	0.830	0.002*	0.791	0.003*

† In the human-machine comparison section, P values were determined to test the differences of AUC, sensitivity or specificity between each pathologist and the nomogram model.
‡ In the human-machine fusion section, P values were determined to test the differences of AUC, sensitivity or specificity that each pathologist achieved before and after the assistance of nomogram.
# P values for the comparison of AUC were determined by Delong's test.
§ P values for the comparison of sensitivity and specificity were determined by McNemar test.
* There was a statistical significance in the comparison with $P < 0.05$.
Abbreviation: AUC, area under receiver-operating characteristic curve; Nom, nomogram; SP, senior pathologist; JP, junior pathologist.

Table S5. Characteristics of patients in clinical cohort

Characteristics[†]	non-myocardial infarction	myocardial infarction	P
n	179	25	
Male (%)	112 (62.6)	20 (80.0)	0.138
Age (year)	62.28±9.75	58.32±10.89	0.062
Body mass index	24.53±3.49	25.16±3.14	0.438
Coronary angiography	164 (91.6)	25 (100)	0.274
Unstable angina pectoris (%)	163 (91.1)	25 (100.0)	0.198
Coronary total occlusion (%)	28 (15.6)	23 (92.0)	<0.001*
Severe stenosis[‡] (%)	67 (37.4)	24 (96.0)	<0.001*
Coronary artery disease (%)	128 (72.3)	25 (100.0)	0.006*
Left ventricular posterior wall thickness (mm)	9.64±1.34	10.32±1.52	0.02*
Right ventricular anterior wall thickness (mm)	4.17±1.05	4.19±1.17	0.92
Diameter of pulmonary annulus (mm)	22.27±2.83	22.10±3.79	0.80

† Characteristics were summarized as frequency with percentage for nominal variable and mean with standard deviation for continuous variable with normal distribution.
‡ Severe stenosis was defined as stenosis level of at least one coronary artery higher than 75%.
* There was a statistical significance in the comparison with P<0.05.

Figure S1. Missingness map of datasets of six forensic centers

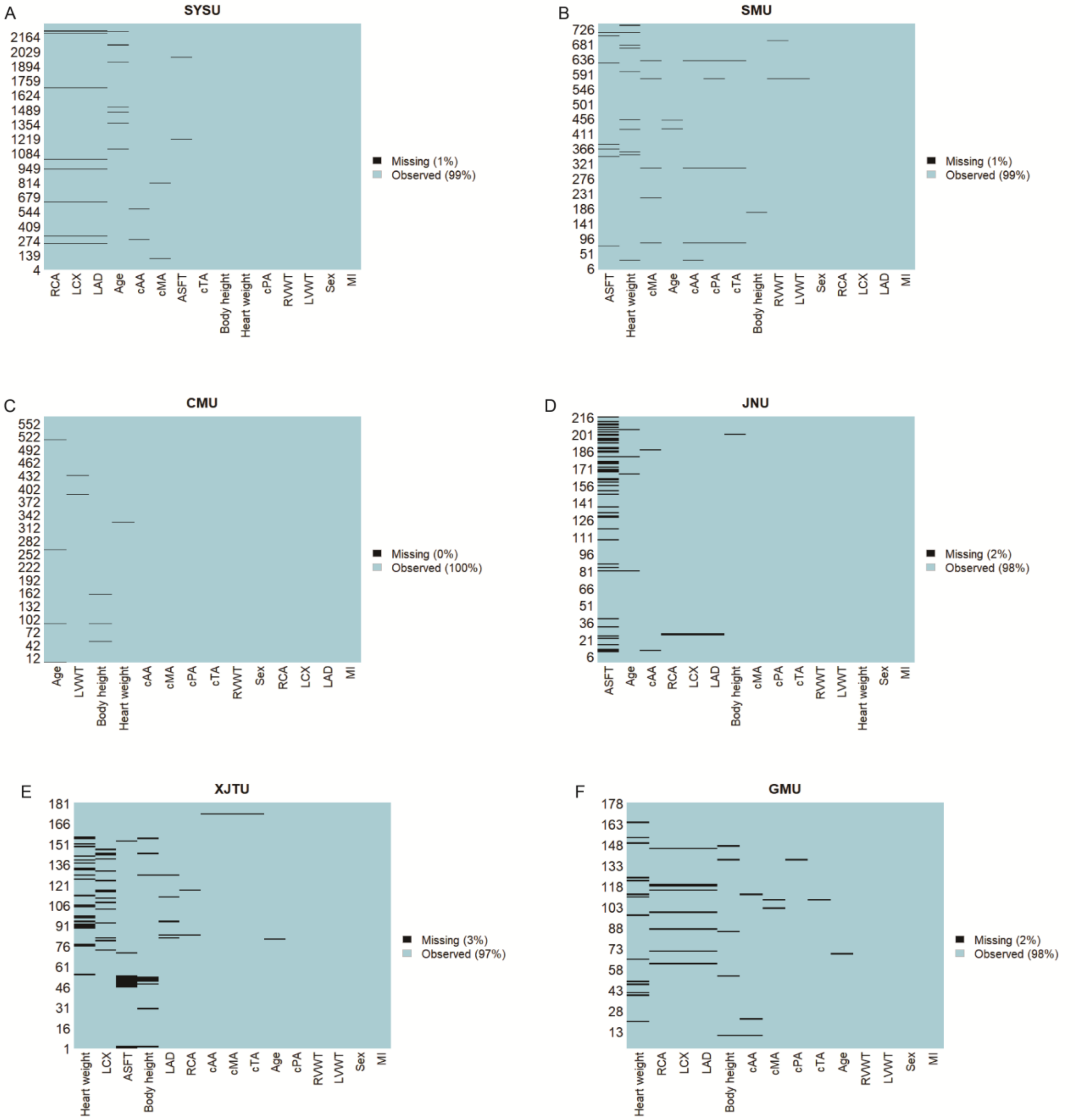

Missingness map of the dataset in Sun Yat-sen University (A), Southern Medical University (B), China Medical University (C), Jinan University (D), Xi'an Jiaotong University (E), and Guizhou Medical University (F).

Figure S2. The correlation heatmap of variables in the interaction of LASSO and RF-RFE method

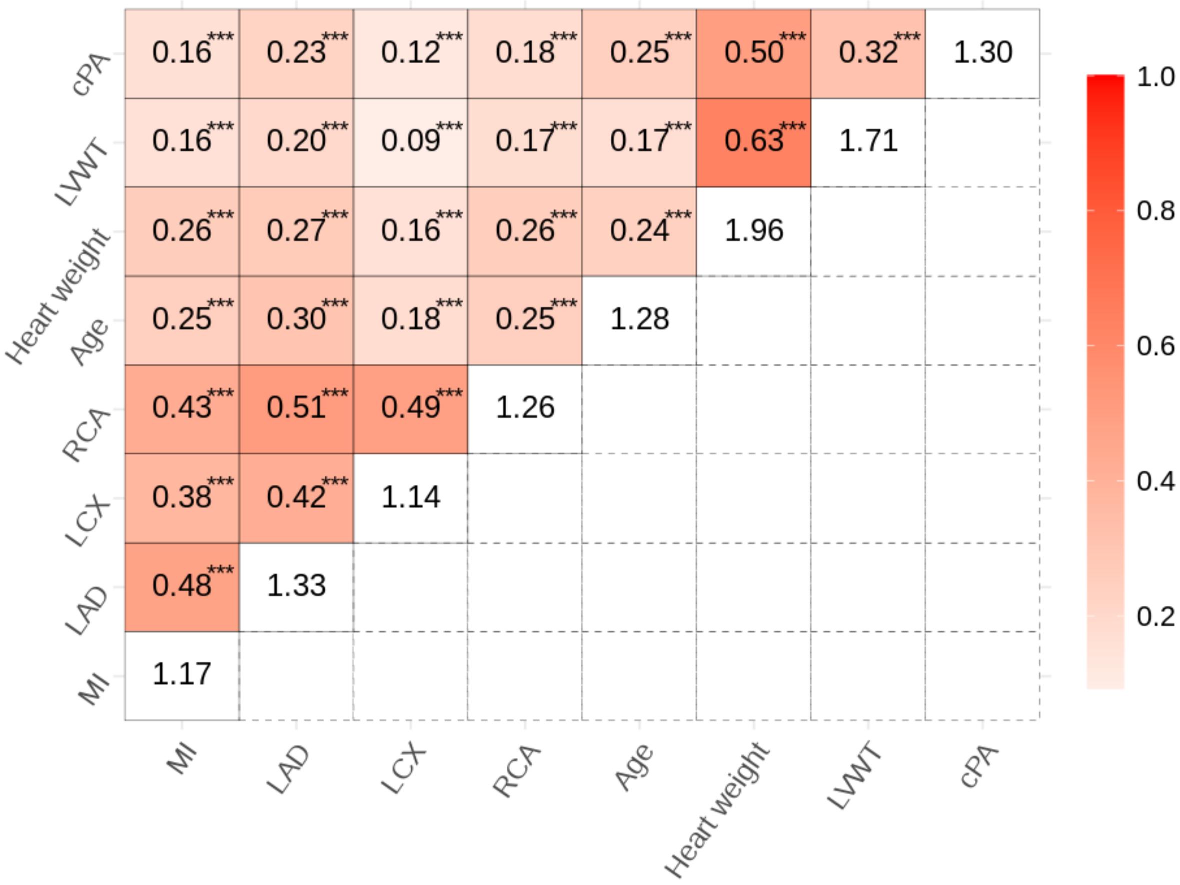

The correlation heatmap of variables in the interaction of LASSO and RF-RFE method. Pearson correlation test, Point-biserial correlation test, and chi-square test with phi (φ) coefficient were utilized to assess the correlation between continuous and continuous variables, between continuous and dichotomous variables, and between dichotomous and dichotomous variables, respectively. The variance inflation factor of each variable is represented on the diagonal line. The ***, **, * symbols on the upper right corner of each grid represent a statistic significance with P<0.001, 0.01, 0.05, respectively. Abbreviation: cPA, circumference of pulmonary annulus; LVWT, left ventricle wall thickness; RCA, right coronary artery; LCX, left circumflex artery; LAD, left anterior descending artery; MI, myocardial infarction.

Figure S3. ROC curves of eight ML models in four external datasets

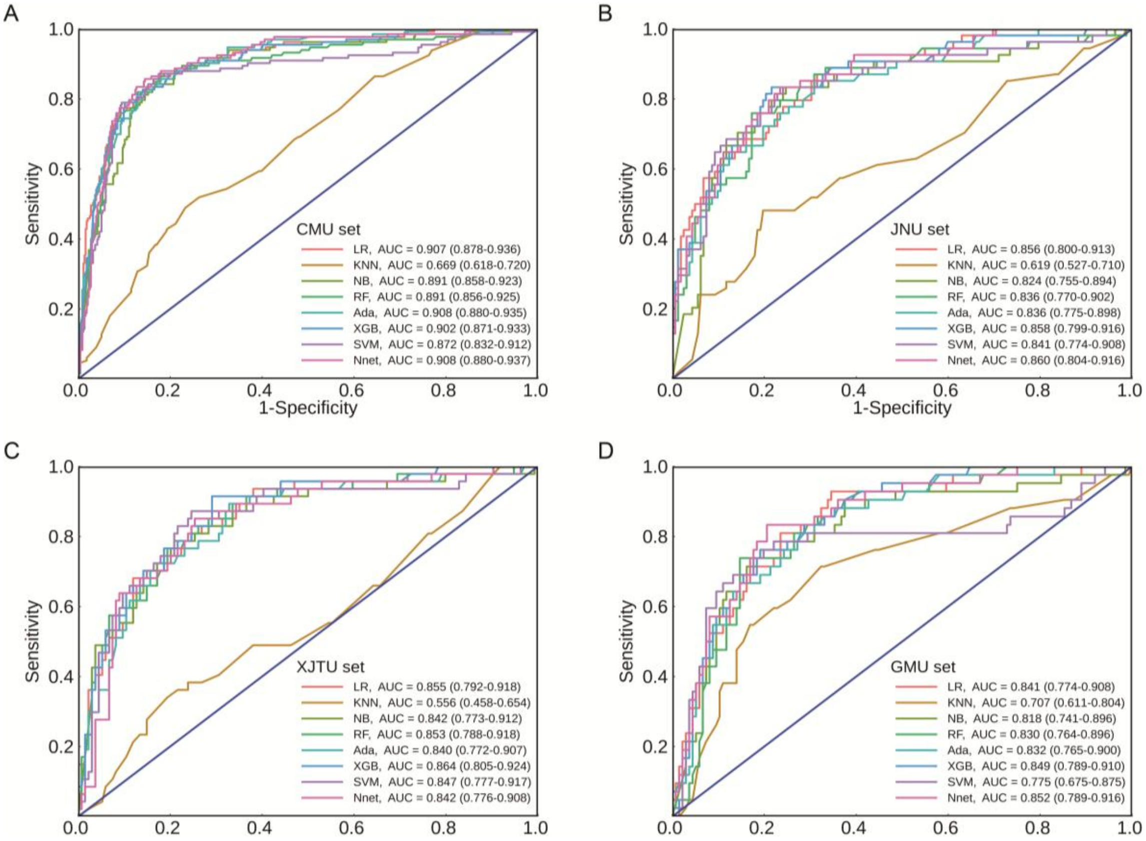

ROC curves of eight ML models in the forensic centers of China Medical University (A), Jinan University (B), Xi'an Jiaotong University (C), and Guizhou Medical University (D). Abbreviation: ML, machine learning.

Figure S4. Sensitivity analysis about the missingness during the model construction

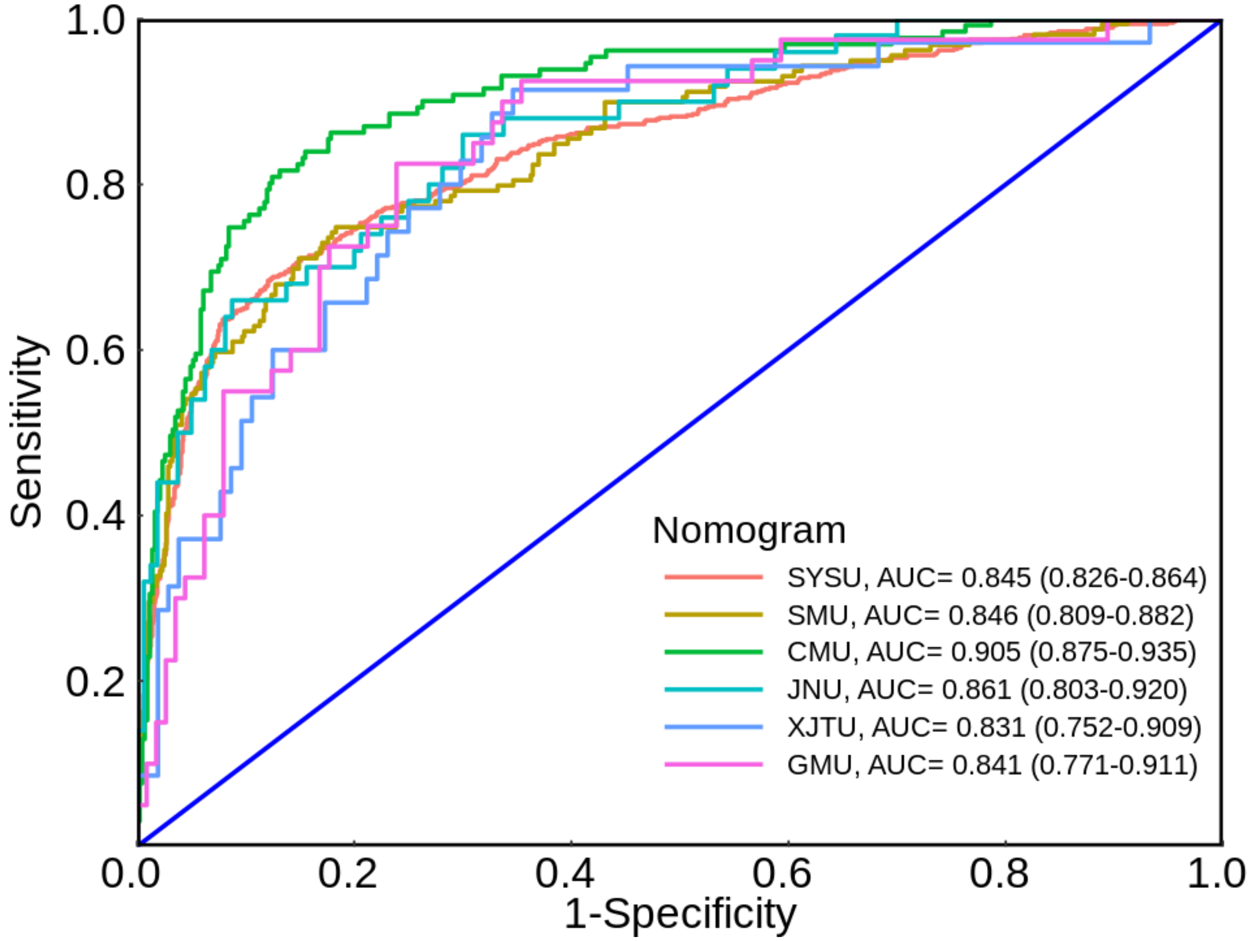

ROC curves of the reconstructed nomogram in the training set and external centers. The nomogram was reconstructed in the training set with cases with missingness excluded (final n=2,170) and validated in external centers with cases with missingness falling in the final candidate set involved in the diagnostic model excluded. Abbreviation: SYSU, Sun Yat-sen University; SMU, Southern Medical University; CMU, China Medical University; JNU, Jinan University; XJTU, Xi'an Jiaotong University; GMU, Guizhou Medical University.

Figure S5. Subgroup analyses of the nomogram in natural death and sudden death mode

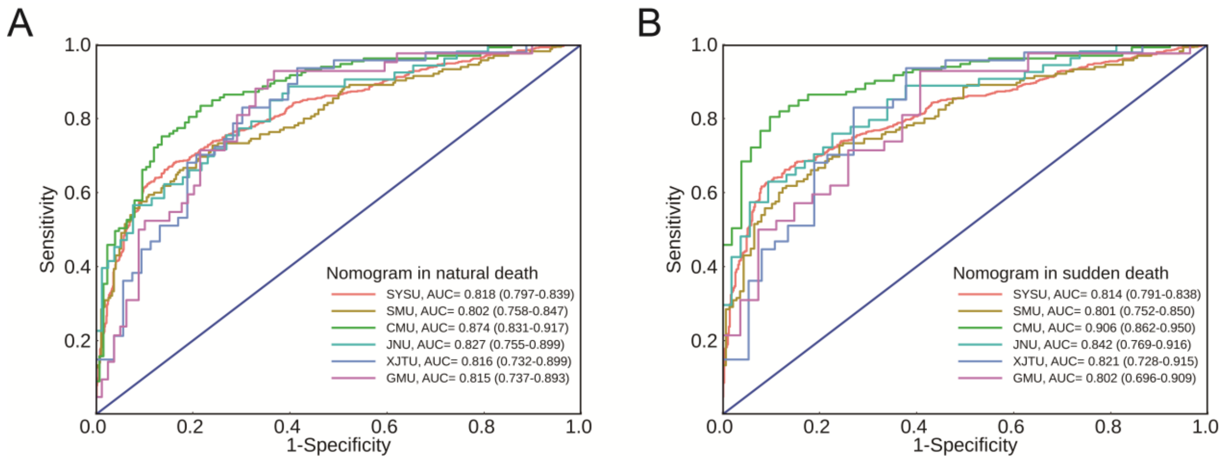

ROC curves of the well-developed nomogram in six datasets for the diagnosis of sudden cardiac death in natural death (A) and sudden death (B) mode.

Figure S6. Subgroup analyses of the nomogram in different diseases

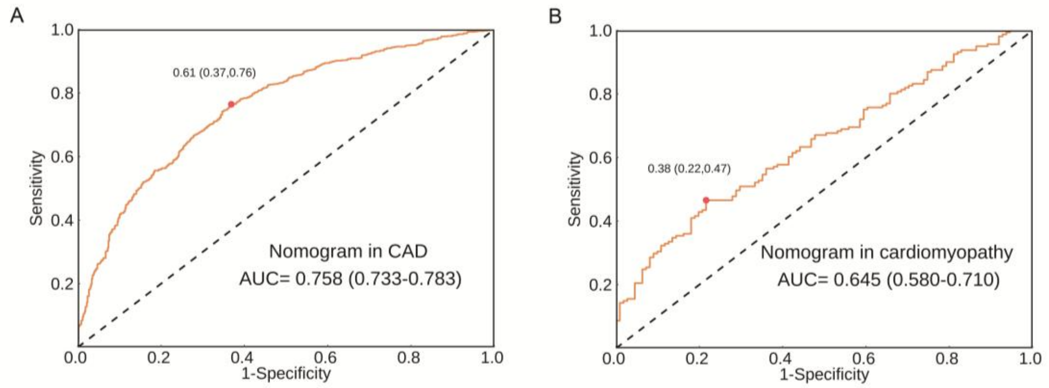

ROC curves of the well-developed nomogram in six datasets for the diagnosis of SCD in coronary artery disease (A) and cardiomyopathy (B). Abbreviation: CAD, coronary artery disease.

Figure S7. Feature selection for the prediction of sudden coronary artery death among individuals with coronary artery disease in forensic setting

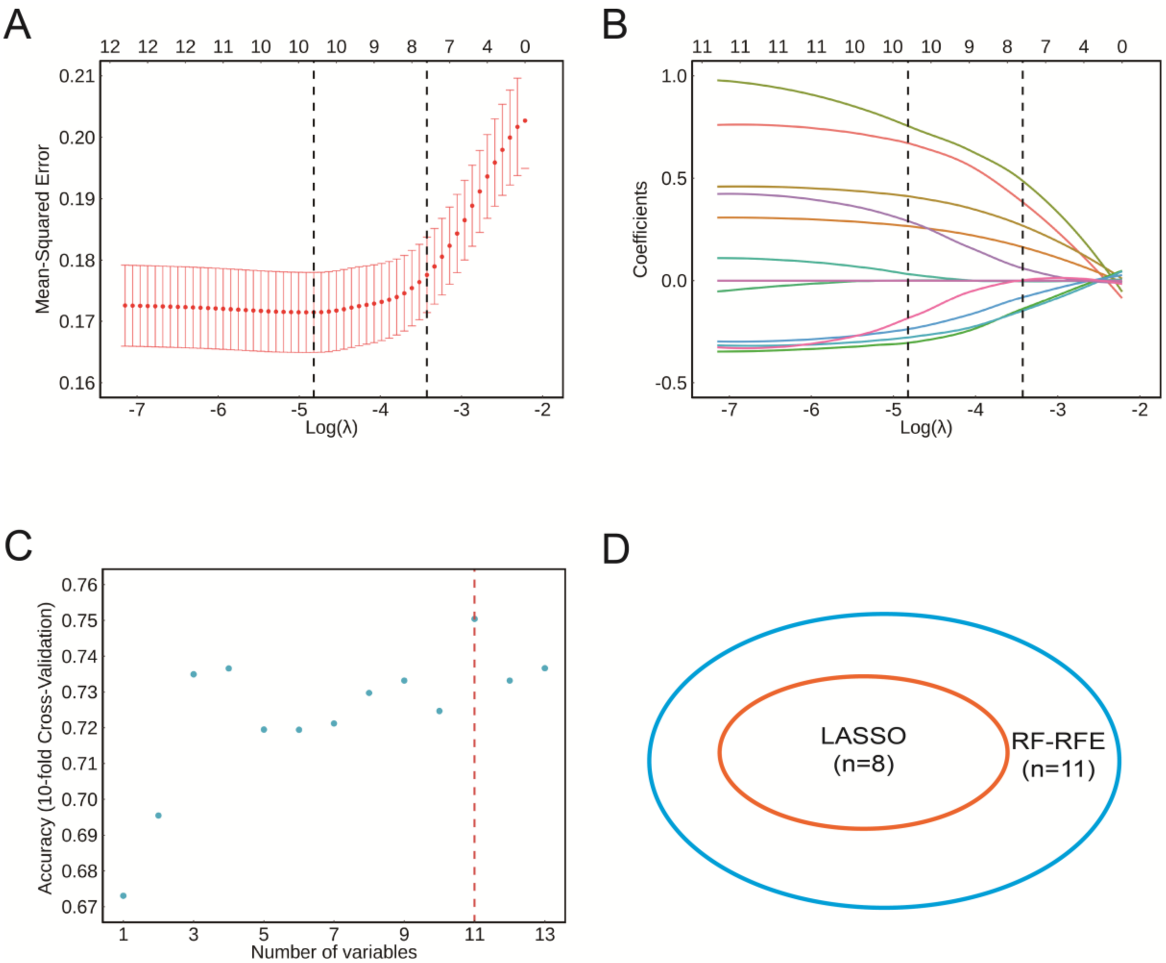

Feature selection and development of logistics model for the prediction of sudden coronary artery death in forensic centers. (A) Plot of mean squared error versus log (λ) in LASSO regression model with 10-fold cross-validation. Dotted vertical lines are drawn at the optimal values by utilizing the minimum criteria (left line) and the 1 standard error criterion (right line, selected as the optimal penalty parameter in this study). (B) LASSO coefficient profiles. Curves represent coefficient profiles of features across regularization parameters (λ), with colors distinguishing individual variables. The optimal λ accounts for eight variables (including age, sex, LAD, LCX, RCA, LVWT, RVWT, and cPA) with eventually non-zero coefficients. (C) Scatter plot of accuracy versus number of variables selected in RF-RFE with 10-fold cross-validation. The red dotted vertical line is drawn at the optimal number of variables (n=11, including age, sex, body height, abdominal subcutaneous fat thickness, LAD, LCX, RCA, LVWT, RVWT, cPA, and cMA) that achieves the highest accuracy. (D) Venn diagram according to the relationship of variables selected by LASSO and RF-RFE method. Abbreviation: LASSO, least absolute shrinkage and selection operator; RF-RFE, recursive feature elimination based on random forest; LAD, left anterior descending artery; LCX, left circumflex artery; RCA, right coronary artery; LVWT, left ventricle wall thickness; RVWT, right ventricle wall thickness; cPA, circumference of pulmonary annulus; cMA, circumference of mitral annulus.

Figure S8. The two-dimensional echocardiography-available measurement of heart morphological features

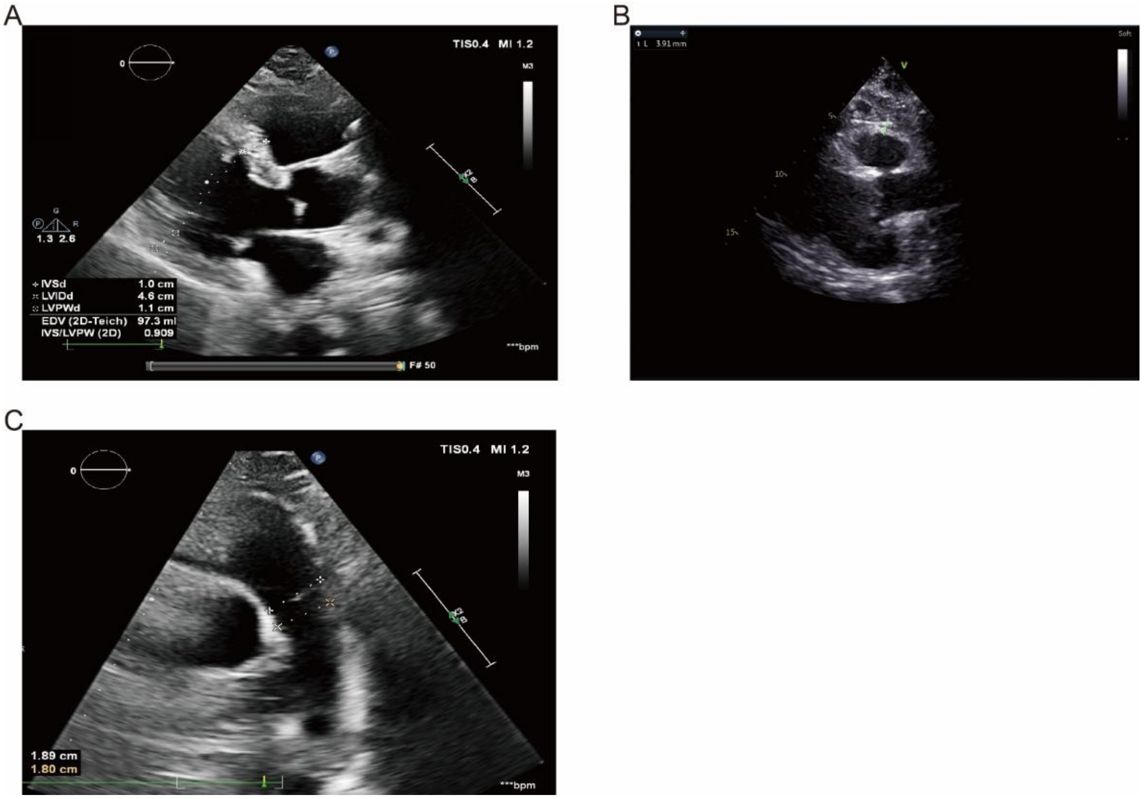

The measurement of thickness of left ventricular posterior wall (**A**) and right ventricular anterior wall (**B**) in parasternal long axis view and the diameter of pulmonary valve (**C**) in pulmonary artery long axis view through two-dimensional echocardiography.
